# McSplicer: a probabilistic model for estimating splice site usage from RNA-seq data

**DOI:** 10.1093/bioinformatics/btab050

**Published:** 2021-01-30

**Authors:** Israa Alqassem, Yash Sonthalia, Erika Klitzke-Feser, Heejung Shim, Stefan Canzar

**Affiliations:** Gene Center, Ludwig-Maximilians-Universität München, Munich 81377, Germany; Dept. of Computer Science, Purdue University, West lafayette 47907, IN, USA"and additionally add"Present Address: Google, Kirkland 98033, WA, USA; Gene Center, Ludwig-Maximilians-Universität München, Munich 81377, Germany; Melbourne Integrative Genomics and School of Mathematics and Statistics, University of Melbourne, Parkville, Victoria 3010, Australia; Gene Center, Ludwig-Maximilians-Universität München, Munich 81377, Germany

## Abstract

**Motivation:**

Alternative splicing removes intronic sequences from pre-mRNAs in alternative ways to produce different forms (isoforms) of mature mRNA. The composition of expressed transcripts gives specific functionalities to cells in a particular condition or developmental stage. In addition, a large fraction of human disease mutations affect splicing and lead to aberrant mRNA and protein products. Current methods that interrogate the transcriptome based on RNA-seq either suffer from short-read length when trying to infer full-length transcripts, or are restricted to predefined units of alternative splicing that they quantify from local read evidence.

**Results:**

Instead of attempting to quantify individual outcomes of the splicing process such as local splicing events or full-length transcripts, we propose to quantify alternative splicing using a simplified probabilistic model of the underlying splicing process. Our model is based on the usage of individual splice sites and can generate arbitrarily complex types of splicing patterns. In our implementation, McSplicer, we estimate the parameters of our model using all read data at once and we demonstrate in our experiments that this yields more accurate estimates compared to competing methods. Our model is able to describe multiple effects of splicing mutations using few, easy to interpret parameters, as we illustrate in an experiment on RNA-seq data from autism spectrum disorder patients.

**Availability and implementation:**

McSplicer source code is available at https://github.com/canzarlab/McSplicer and has been deposited in archived format at https://doi.org/10.5281/zenodo.4449881.

**Supplementary information:**

Supplementary data are available at *Bioinformatics* online.

## 1 Introduction

Through alternative splicing (AS), a single gene can produce multiple mRNA transcripts, or isoforms, that combine exons in alternative ways. Approximately 95% of human multi-exon protein-coding genes undergo alternative splicing (Pan *et al.*, 2008), creating a remarkably complex set of transcripts that give specific functionalities to cells and tissues in a particular condition or developmental stage.

**Fig. 1.**
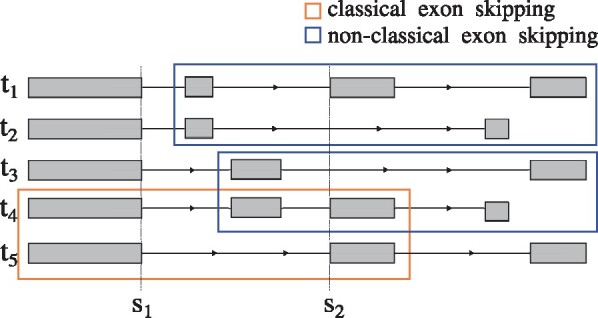
Complex alternative splicing involving five different transcripts. The two classical exon skipping events between *t*_1_ and *t*_5_, and between *t*_4_ and *t*_5_ do not fully capture the overall complexity. The two exon skippings marked in blue are not considered classical events and would not be reported by methods such as SplAdder, since they also differ in the last exon. Methods such as MAJIQ generalize simple events to more complex AS units that contain all introns sharing a common splice site. Two such AS units are required to describe the simple exon skipping event marked in orange, one comprising three introns sharing donor *s*_1_ and one containing three different introns sharing acceptor *s*_2_

RNA sequencing (RNA-seq) is routinely used in genome-wide transcript analysis. This technology produces short reads from which existing methods infer and quantify RNA splicing, broadly, in one of two different ways. Methods either analyze full-length transcripts or focus on individual splicing events. Transcript assembly methods such as StringTie (Pertea *et al.*, 2015), CIDANE (Canzar *et al.*, 2016) and CLASS (Song and Florea, 2013) aim to identify the set of expressed full-length transcripts which in principle provides a complete picture of all splicing variations, see e.g. transcript *t*_1_–*t*_5_ inFigure 1. The transcript assembly problem is, however, ill-posed (Lacroix *et al.*, 2008) and error-prone especially for complex genes expressing multiple transcript isoforms (Hayer *et al.*, 2015). Event-based methods, therefore, focus on local splicing patterns such as the classical exon skipping event denoted in Figure 1, without a prior attempt to assemble or quantify full-length transcripts. The relative abundance of different splicing outcomes that can potentially be shared by multiple transcripts, can then be quantified using a simple metric such as percent spliced in (PSI) (Venables *et al.*, 2008). A notable exception is SUPPA (Alamancos *et al.*, 2015) which derives PSI values from quantified transcript abundances.

Event-based methods differ in the complexity of the units of AS they quantify. In the simplest case, methods such as MISO (Katz *et al.*, 2010), SUPPA, ASGAL (Denti *et al.*, 2018), SpliceGrapher (Rogers *et al.*, 2012) and SplAdder (Kahles *et al.*, 2016) identify one of the canonical types of AS, such as exon skipping, alternative 5′ and 3′ splice sites, intron retentions and mutually exclusive exons (see Supplementary Fig. S1). In Figure 1, this definition would include the two simple exon skippings between *t*_1_ and *t*_5_ and between *t*_4_ and *t*_5_, and mutually exclusive spliced exons in *t*_1_ and *t*_4_, clearly underestimating the full AS complexity across *t*_1_–*t*_5_.

Compared to these simple types of splicing events, complex events involve multiple alternative splice sites or exons and according to Vaquero-Garcia *et al.* (2016) constitute at least one-third of AS events observed in human and mouse tissues. Methods such as JUM (Wang and Rio, 2018), MAJIQ (Vaquero-Garcia *et al.*, 2016) and the method proposed in Oesterreich *et al.* (2016), therefore consider AS units that generalize simple events to more complex patterns. They quantify the relative usage of an arbitrary number of introns that share a common splice site. Since these AS units capture only the common endpoints of alternative splicing patterns, such methods need to quantify two AS units for a single exon skipping event (Fig. 1). LeafCutter (Li *et al.*, 2018) and Whippet (Sterne-Weiler *et al.*, 2017) add further introns to AS units. At the extreme end, Whippet enumerates all possible transcript fragments that combine overlapping events and estimates their relative abundance using an EM algorithm similar to full-length transcript quantification methods such as kallisto (Bray *et al.*, 2016).

PSGInfer (LeGault and Dewey, 2013) quantifies alternative splicing based on Probabilistic Splice Graphs (PSGs). It assigns weights to the edges of a splicing graph (Heber *et al.*, 2002) using parameters that describe the splicing process, rather than focusing on individual outcomes of the splicing processes such as local splicing events or full-length transcripts. The parameter estimates can then be used to estimate transcript and processing event frequencies. Motivated by the work by LeGault and Dewey (2013), we similarly aim to quantify alternative splicing by building a probabilistic model as a simple approximation to the underlying splicing processes. In constrast to PSG, however, our model employs the usages of annotated as well as novel splice sites across all expressed transcripts to describe a simplified splicing process that has generated the set of expressed transcripts. Traversing the linear ordering of all exons of a gene from 5′ to 3′, the usage of each splice site specifies the probability with which the site is used as donor or acceptor site. For example, the usage of acceptor *s*_2_ in Figure 1 indicates the abundance of transcripts *t*_1_, *t*_4_ and *t*_5_ that ‘use’ the acceptor relative to the total output *t*_1_–*t*_5_ of the gene. Our model assumes that splice site usages are independent of each other, which allows for a computationally more efficient estimation of parameters compared to PSGInfer.

This model by definition can generate complex splicing patterns that do not rely on any predefined simple or complex AS units as event-based methods like SplAdder, MAJIQ or LeafCutter do. At the same time, splice site usages that capture simultaneous changes in multiple isoforms facilitate the interpretation of point mutations that disrupt splicing as is the case in many genetic disorders (Anna and Monika, 2018). Instead of attempting to quantify each one of multiple possible effects on intron or even transcript level, a reduced splice site usage as computed by McSplicer may directly reflect the weakening of a splice site by a point mutation in the consensus splice site sequence that is responsible for these effects, as we illustrate in our experiments on RNA-seq data from autism spectrum disorder patients (Section 3.4).

Furthermore, our method simultaneously estimates the model parameters, i.e. splice site usages, using all reads mapped to a gene locus, often resulting in more accurate estimates compared to event-based methods that use only reads directly supporting their parameters. We demonstrate the improved accuracy of McSplicer compared to existing methods in our experiments.

## 2 Materials and methods

**Fig. 2.**
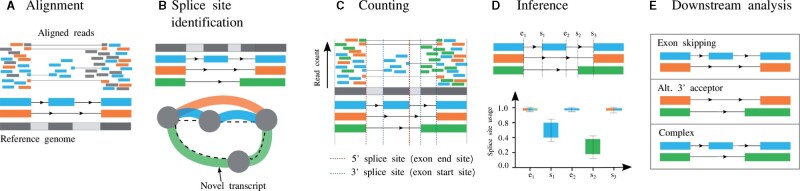
McSplicer workflow summary. The main steps of the McSplicer analysis are: (**A**) Map RNA-seq reads to the reference genome sequence. (**B**) Identify annotated as well as novel splice sites through the reference-based assembly of transcripts using, e.g. StringTie (Pertea *et al.*, 2015). (**C**) Divide the gene into non-overlapping segments bounded by splice sites, TSS and TES and count the number of reads mapping to distinct combinations of segments. In this example, only the start of the first exon and the end of the last exon are bounded by TSS and TES, respectively, the remaining exon start and end sites correspond to splice sites. (**D**) Estimate splice site usages using McSplicer. (**E**) Leverage splice site usages in various kinds of downstream analyses, such as the quantification of different types of alternative splicing events

A typical RNA-seq analysis workflow that uses McSplicer to estimate the usage of splice sites consists of the five steps illustrated in Figure 2. After (A) mapping reads in an RNA-seq sample to a reference genome sequence using a read alignment tool such as STAR (Dobin *et al.*, 2013) or HISAT (Kim *et al.*, 2015), we (B) assemble reads to full-length transcripts using methods such as StringTie (Pertea *et al.*, 2015) or CLASS (Song and Florea, 2013) to identify annotated as well as novel splice sites. Step (B) can be omitted and instead a curated catalog of known transcripts may be provided. In both cases, McSplicer does not rely on any transcript-level phasing of exons but uses the extracted splice sites and transcription start (TSS) and end sites (TES) to (C) partition a gene into contiguous, non-overlapping segments. Segments are defined as minimal subsequences of a gene’s exons and introns that are bounded by splice sites, TSS or TES. The example shown in Figure 2C contains six such segments. We count reads that overlap distinct combinations of such segments. The precise sequence of segments a mapped read overlaps defines its mapping *signature* (Canzar *et al.*, 2016). Reads that map to the same signature are equivalent in terms of the splicing pattern they represent. From *signature counts*, i.e. the number of reads mapping to the same signature (see Supplementary Fig. S2 for an illustration), McSplicer estimates splice site usages in step (D). Splice site usages computed by McSplicer can be leveraged in (E) different types of downstream anlyses, including the quantification of various types of splicing events.

In the following sections, we introduce McSplicer’s model and algorithm for the estimation of parameters in that model. A more detailed description of the model and algorithms is provided in Supplementary Section S2. In the technical description of our model, we refer to the exon boundaries at the 3′ (acceptor) splice site and at the TSS as exon start sites, and to the 5′ (donor) splice sites and TES as exon end sites. The description of our model is based on single-end reads which we apply to paired-end reads in Section 3.3. In the next section, we recapitulate the commonly assumed generative model of RNA-seq that also underlies the McSplicer model. For the sake of simplicity, we introduce the model based on individual observed reads and explain how parameters can be estimated from (much fewer) signature counts at the end of Section 2.3.

### 2.1 A generative model for RNA-seq reads

Consider the RNA-seq reads that mapped to a given gene. Reads are derived from one end of each of *N* fragments and each read has length *L*. We assume that each fragment is independently generated from one of the possible transcripts allowed by our model (see next Section). In this section, we describe a generative model for the sequence of the *n*th read *R_n_*. The probability of *R_n_* can be written as

(1) $$P(R_{n})=\sum_{t}P(R_{n}|T_{n}=t)P(T_{n}=t),$$

where *T_n_* represents the transcript from which *R_n_* was generated. Following models in Li *et al.* (2010) and LeGault and Dewey (2013), we assume that the probability of generating *R_n_* from a transcript *t* is proportional to the product of the (effective) length of the transcript, *l*(*t*), and the relative abundance of the transcript, *w*(*t*):

(2) $$P(T_{n}=t)=\frac{l(t)w(t)}{\sum_{t\prime }l(t\prime )w(t\prime )}.$$

The effective length of a transcript denotes the number of possible start position of a sampled read (Trapnell *et al.*, 2010). We introduce *B_n_* that denotes the start position of *R_n_* in *T_n_*, leading to

(3) $$P(R_{n}|T_{n}=t)=\sum_{b=1}^{l(t)}P(R_{n}|B_{n}=b,T_{n}=t)P(B_{n}=b|T_{n}=t).$$

Making the simplifying assumption that *R_n_* was generated uniformly across transcript *t*, we have

(4) $$P(B_{n}=b|T_{n}=t)=\frac{1}{l(t)}.$$

$P(R_{n}|B_{n}=b,T_{n}=t)=1$ if *R_n_* is identical to the sequence of length *L* starting at a position *b* in transcript *t*, and this probability is 0 otherwise.

### 2.2 McSplicer: an inhomogeneous Markov chain to model the relative abundance of transcripts

**Fig. 3.**
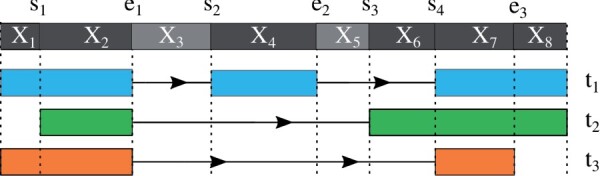
Example of hidden states representing three different transcripts. Five exon start sites and four exon end sites divide the gene into eight segments. Note, however, that the TSS bounding *X*_1_ from the left and the TES bounding *X*_8_ from the right are not labeled here since our model treats them differently (see main text). Therefore, *M_s_* = 4, *M_e_* = 3 and *M *=* *8. The three sequences of states of *Z*, (1, 1, 0, 1, 0, 0, 1,1), (0, 1, 0, 0, 0, 1, 1,1) and (1, 1, 0, 0, 0, 0, 1,0), represent the three transcripts *t*_1_, *t*_2_ and *t*_3_, respectively

We propose a new model for the relative abundance of transcripts expressed by a gene, denoted by *w*(*t*) in the previous section. Suppose we have obtained in step (B) in the McSplicer workflow (Fig. 2) exon start sites, $s_{1},\ldots ,s_{M_{s}}$ and exon end sites, $e_{1},\ldots ,e_{M_{e}}$ ordered by their occurrence in forward direction of a given gene. Here, we do not include the start site of the first exon and the end site of the last exon, since the former is treated differently in our model (see below) and the usage of the latter is always equal to 1 in our model. All exon start and end sites partition the gene into non-overlapping segments $X_{1},\ldots ,X_{M}$, where $M=M_{s}+M_{e}+1$ and each segment is defined by a region enclosed by splice sites or transcription start or end sites that occur consecutively along the genome (see Figs 2C and 3). We introduce a sequence of hidden variables, $Z=(Z_{1},\ldots ,Z_{M})$, where *Z_i_* is a binary indicator for whether the *i*th segment *X_i_* is transcribed (*Z_i_* = 1). Then, a particular transcript can be represented by a sequence of states for *Z*, as illustrated for transcripts $t_{1},t_{2},t_{3}$ in Figure 3. Thus, we can model the relative abundance of transcripts by modeling the probability of *Z*.

We use an inhomogeneous Markov chain to model the probability of the sequence of hidden variables, $Z=(Z_{1},\ldots ,Z_{M})$. Specifically, the initial probability is given by

(5) $$P(Z_{1}=1)=\pi ,$$

where *π* represents the proportion of transcripts that contain the first segment. We model the transition probability from *Z_i_* to $Z_{i+1}$ for i=1,…,M−1 as follows. If two consecutive segments *X_i_* and $X_{i+1}$ are separated by an exon start site *s_m_*,

(6) $$P(Z_{i+1}=1|Z_{i}=0)=p_{m}$$

 

(7) $$P(Z_{i+1}=1|Z_{i}=1)=1.$$

If they are separated by an exon end site *e_m_*,

(8) $$P(Z_{i+1}=0|Z_{i}=1)=q_{m}$$

 

(9) $$P(Z_{i+1}=0|Z_{i}=0)=1.$$

That is, if the current segment is transcribed (*Z_i_* = 1), the splicing process ignores an exon start site (Equation 7), but it considers the potential usage of an exon end site *e_m_* and decides to use it, i.e. end the exon, with its usage probability *q_m_* (Equation 8). On the other hand, if the current segment is not transcribed (*Z_i_* = 0), the splicing process ignores an exon end site (Equation 9), but it uses an exon start site *s_m_* with its usage probability *p_m_* (Equation 6). The parameters $P(Z_{i+1}=0|Z_{i}=0)=1.$ and $q=(q_{1},\ldots ,q_{M_{e}})$ represent probabilities of using the corresponding exon start and end sites, respectively, given that each site is considered for potential usage. Throughout the rest of this work, we refer to these usage probabilities simply as usages. Supplementary Table S1 shows the relative abundances defined by the proposed model for the three transcripts presented in Figure 3. A more detailed description is provided in Supplementary Sections S2.1–S2.3.

### 2.3 Parameter estimation and uncertainty quantification

We use an EM algorithm to compute the maximum likelihood estimates for the model parameters Θ={π,p,q}, that is $\hat{\Theta }:={\text{argmax}}_{\Theta }P(R_{1},\ldots ,R_{N}|\Theta )$. The complete log likelihood in the EM algorithm involves $P(R_{n},B_{n}=b,T_{n}=Z|\Theta )$ for b∈{1,…,l(Z)} (Supplementary Section S2.4). By combining the generative model and the McSplicer model in the previous two sections, $P(R_{n},B_{n}=b,T_{n}=Z|\Theta )$ can be written as

(10) $$\begin{array}{cc} & P(R_{n}|B_{n}=b,T_{n}=Z)P(B_{n}=b|T_{n}=Z)P(T_{n}=Z|\Theta )\\ & =\frac{1}{l(Z)}\frac{l(Z)w_{\Theta }(Z)}{\sum_{Z\prime }l(Z\prime )w_{\Theta }(Z\prime )}=\frac{w_{\Theta }(Z)}{\sum_{Z\prime }l(Z\prime )w_{\Theta }(Z\prime )} \end{array}$$

if *R_n_* is identical to the sequence of length *L* starting at position *b* in transcript *Z*. Otherwise, this probability is 0. The details of the application of the EM algorithm to the proposed model are provided in Supplementary Section S2.4. The EM algorithm uses several quantities that we compute using dynamic programming, see Supplementary Section S2.5. Also, all quantities required in our EM algorithm can be computed using only signature counts (Supplementary Section S2.4), so the input to McSplicer are the signature counts rather than individual reads.

We quantify the uncertainty of our estimator $\hat{\Theta }$ using bootstrapping. Specifically, let $c={\left(c_{j}\right)}_{j=1}^{J}$ represent the signature counts over *J* signatures defined for a given gene, where the total signature count equals the total read count in the gene, i.e. $\sum_{j=1}^{J}c_{j}=N$. We draw *B* independent bootstrap samples, $c^{1},\ldots ,c^{B}$, from a multinomial distribution:

(11) $$c^{b}∼\text{multinomial}(\frac{c_{1}}{N},\ldots ,\frac{c_{J}}{N},N).$$

Then, we compute *B* bootstrap estimators, ${\hat{\Theta }}^{1},\ldots ,{\hat{\Theta }}^{B}$, by applying our EM algorithm to each bootstrap sample and use them to approximate the sampling distribution of our estimator $\hat{\Theta }$. In this paper, we quantify the uncertainty of $\hat{\Theta }$ using a confidence interval computed from the approximated sampling distribution. Other types of uncertainty quantification could easily be obtained from the bootstrap estimators.

### 2.4 Simulated datasets and evaluation

We used Polyester (Frazee *et al.*, 2015) to simulate reads from a human transcriptome with abundances estimated from a real RNA-experiment (GEO accession GSM3094221) using RSEM (Li and Dewey, 2011). Based on these ground truth expressions, we simulated datasets with varying sequencing depth commonly observed in practice, including 20 million, 50 million and 75 million reads of 100 bp length. Following the same strategy as Soneson *et al.* (2016), we randomly selected a set of 1000 genes with at least two expressed transcripts and sufficiently high ground truth expression (gene-level read count per kilobase above 500). Among splice sites for which parameters estimated by compared methods have the same meaning (*comparable* splice sites, introduced in Section 3), we exclude from the analysis constitutive ones with true usage 1 and splice sites that are not used by any of the expressed transcripts (usage 0). That is, only splice sites that are alternatively used by expressed transcripts are considered.

From the ground truth abundance of transcripts, we calculate the true usage of a splice site as the relative contribution of transcripts using a given splice site to the total expression of a gene (see Supplementary Section S2.6.3). We quantify the accuracy of splice site usages inferred by each method by using the Kullback-Leibler (KL) divergence, defined in Supplementary Section S2.6.4. All code and data necessary to reproduce the results of this simulation study are available at https://github.com/canzarlab/McSplicer.

## 3 Results

We assess the performance of McSplicer in comparison to existing state-of-the-art methods on both simulated and real RNA-seq datasets. Simulated data allow to compare estimates to a known ground truth of expressed transcripts and thus known quantities of alternative splicing events. On the other hand, simulated data cannot fully capture the complexity of datasets generated in real RNA-seq experiments. Note that exon start and end sites whose usage McSplicer estimates can correspond to splice sites but also to transcription start and end sites (see Section 2.2). In the following, however, we restrict the evaluation to the usage of splice sites since transcription start and end sites cannot be reliably estimated from short-read RNA-seq data alone.

We compare the performance of McSplicer to PSGInfer, SplAdder, MAJIQ and StringTie. In Supplementary Section S2.6.1 we provide details on software versions and command line arguments used. PSGInfer quantifies alternative splicing using a generative probabilistic model, an idea that also motivated the approach taken in McSplicer. SplAdder was used in a large-scale study (Kahles *et al.*, 2018) to detect and quantify alternative splicing events in nearly 9000 tumor RNA-seq samples. In a comparative benchmark analysis performed in Kahles *et al.* (2016) it showed a better performance than competing methods JuncBase (Brooks *et al.*, 2011), rMATS (Shen *et al.*, 2014) and SpliceGrapher (Rogers *et al.*, 2012), from which, of course, general superiority cannot be concluded (Denti *et al.*, 2018). Compared to SplAdder, which is limited to the detection of simple types of splicing events, MAJIQ introduced a novel approach that additionally captures more complex transcript variations. MAJIQ was shown in a recent benchmark (Mehmood *et al.*, 2020) to compare favorably to existing state-of-the-art methods and the authors demonstrated in Vaquero-Garcia *et al.* (2018) that MAJIQ also outperforms LeafCutter and rMATS.

StringTie, on the other hand, assembles and quantifies full-length transcripts from RNA-seq but was not specifically designed for the quantification of splice site usage. Nevertheless, splice site usage can be inferred from the abundance of the assembled transcripts and we include this approach as a baseline in our benchmark: In all experiments, McSplicer uses StringTie to construct the exon-intron structure in steps (B) and (C) of the workflow (Fig. 2), which potentially contains novel splice sites. In contrast to the inference of splice site usage from expressed full-length transcripts, however, McSplicer estimates the usage of the same set of splice sites using the EM algorithm described in the previous section.

Each method, however, uses a different set of parameters to quantify alternative splicing events. PSGInfer infers the weights of its constructed splice graph edges. SplAdder quantifies four canonical types of splicing events using the widely used *percent spliced in* (PSI) metric. PSI denotes the ratio between the number of reads supporting one outcome of the event (e.g the inclusion of an exon) over the number of reads directly supporting either of the two alternative outcomes. Similarly, MAJIQ computes the *percent selected index* (Ψ) for each splice junction involved in a *local splicing variation* (LSV), which denotes its fractional usage. To ensure a meaningful comparison of splice site usages in McSplicer to edge weights from PSGInfer, PSI from SplAdder and Ψ from MAJIQ, we only consider splice sites for which the meaning of these four quantities, if defined, coincide. These *comparable* splice sites are obtained from alternative splicing events between two expressed transcripts such that all remaining transcripts expressed by a gene consistently support one of the two possible outcomes of the event. Note that comparable splice sites are defined based on transcripts expressed in a given sample. We define comparable splice sites more formally in Supplementary Section S2.6.2. For comparable splice sites of simple events, the four different parameters, i.e. splice site usage, edge weights, PSI and Ψ, equally reflect the relative abundance of transcripts expressed by a given gene that use the splice site, or equivalently contain the corresponding exon. Analogously, Ψ, edge weight and splice site usage are equivalent for comparable splice sites of complex events. We will therefore consistently refer to these different parameters in the following as splice site usage. From StringTie assemblies of full-length transcripts, estimates of splice site usage can directly be obtained from the relative abundance of transcripts using a given splice site. For an illustrative example of comparable and non-comparable splice sites see Supplementary Figure S3.

### 3.1 McSplicer more accurately infers splice site usage than competing methods

In this section, we assess the performance of McSplicer on RNA-seq datasets simulated as described in Section 2.4. All methods but PSGInfer were provided the same set of reads aligned using STAR (allowing mismatches and indels). PSGInfer only accepted unaligned reads which were internally mapped using Bowtie (Langmead *et al.*, 2009). We distinguish splice sites by the type of event they are part of, including exon skipping, intron retention, alternative 3′ and 5′ splice sites, and complex events that cannot be assigned to one of the canonical types. The events are labeled by Astalavista (Foissac and Sammeth, 2007) through a pairwise structural comparison of all transcript species expressed in our ground truth transcriptome (see Supplementary Figs S1 and S4).

The number of variable splice sites (i.e. 0<usage<1) in our simulated dataset, and the number of comparable splice sites among them (∼ 36%), with corresponding event types defined by Astalavista are listed in Supplementary Table S2. It also lists the total number of (comparable) splice sites per type reported by all four methods. While McSplicer will quantify the usage of all splice sites except those missed by StringTie in step (B) in Figure 2, competing methods report only events that satisfy an adjustable confidence threshold (SplAdder) or are considered reliable according to internal filters (MAJIQ). As a result, both MAJIQ’s and SplAdder’s accuracy is evaluated on a smaller, presumably more confidently estimated set of events (Supplementary Table S2) and are otherwise not penalized for missing events. MAJIQ estimates two parameters that correspond to the relative usage of a skipped exon, one based on the intron connecting it to the upstream exon, and one based on the downstream exon (Supplementary Fig. S5). Here, we compare the performance to the latter one, which we observed to be slightly more accurate. The former is reported in Supplementary Figure S6.

**Fig. 4.**
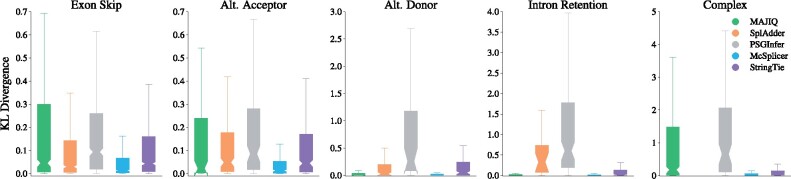
Accuracy of McSplicer and competing methods in quantifying the usage of variable splice sites from 50 million simulated RNA-seq reads. For each method, only splice sites in events that the method reports and quantifies are considered. SplAdder is limited to the quantification of simple AS events

Figure 4 compares the accuracy of splice site usages inferred by McSplicer and competing methods from 50 million reads on four canonical types of events as well as on complex events. For each method, only events reported and quantified by that method are considered. Supplementary Figure S7 shows consistent results when considering events that McSplicer and competing methods have pairwise in common. Across all types of events, McSplicer infers splice site usages more accurately than competing methods. The accuracy of splice site usage inferred by McSplicer is not affected by the complexity of the event, whereas MAJIQ’s estimates are substantially less accurate for complex events. SplAdder is restricted to the quantification of simple events. As originally reported by the authors in Kahles *et al.* (2016), SplAdder quantifies intron retentions less accurately than other simple types of events. Other methods, including McSplicer, perform well on this type of event, which plays an important role for cell development in mammals (Braunschweig *et al.*, 2014) and is a source of neoepitopes in cancer (Smart *et al.*, 2018). We note that different read alignments used in PSGInfer cannot be excluded as a potential contributor to its overall low accuracy. Compared to baseline splice site usage extracted from StringTie transcript assemblies, McSplicer utilizes StringTie’s transcript models to substantially refine the quantification of local splicing variation. We would like to point out, however, that StringTie was designed to assemble full-length transcripts. The comparison to StringTie merely highlights the necessity of additional computations to obtain more accurate estimates of splice site usage. Similar results were obtained on datasets comprising 20 million and 75 million reads (see Supplementary Figs S8 and S9). Furthermore, we demonstrate in Supplementary Figure S10 that McSplicer also achieves accurate estimates on the more challenging set of non-comparable splice sites. While KL divergences are slightly higher than on comparable splice sites, its estimates remain more accurate compared to competing methods that are evaluated only on a subset of comparable splice sites.

Supplementary Figure S11 shows running times of all methods on the three simulated datasets. The splicing model underlying McSplicer allows a much faster estimation of parameters than PSGInfer (∼1 h versus 7 h for 50 million reads), the only other method that is based on a probabilistic model of the splicing process. MAJIQ similarly required around 1 h. As expected, the computation of read count ratios makes SplAdder the fastest method among direct competitors (<14 min). StringTie is by far the fastest method (<3 min), albeit solving a different task. Peak memory usage was below 3 GB for all methods except PSGInfer, which however included as the only method the read mapping step (Supplementary Fig. S12).

### 3.2 McSplicer leverages all reads mapped to a gene

**Fig. 5.**
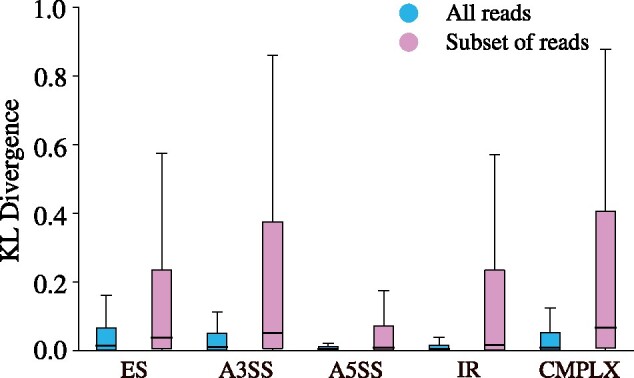
McSplicer leverages all RNA-seq reads mapped to a gene to improve the accuracy of splice site usage estimates. On the dataset with 50 million simulated reads, McSplicer achieves lower KL divergence from true splice site usages when considering all reads mapped to a gene locus at once (blue) compared to using only reads that overlap any of the event’s exons (pink). ES denotes exon skipping, A3SS alternative 3′ splice site, A5SS alternative 5′ splice site, IR intron retention and CMPLX complex events

McSplicer makes use of all reads mapped to a given gene to simultaneously infer parameters in the McSplicer model, while other methods except PSGInfer typically use only reads that directly support their parameters. To quantify the contribution of the simultaneous inference in McSplicer to improve the accuracy of estimators, we estimate one splice site usage parameter at a time using only reads directly supporting the parameter. Similar to the calculation of the traditional PSI metric, we remove for each event with comparable splice sites all reads that do not overlap any of the event’s exons, and run and evaluate McSplicer on the resulting restricted instance as described in the previous section. Figure 5 confirms that McSplicer profits enormously from transcriptional evidence that lies outside of the local splicing event. Across all types of events, McSplicer estimates splice site usage less accurately when reads that do not overlap an event are removed.

### 3.3 McSplicer estimates agree with spike-in RNA variants

To evaluate the performance of McSplicer under the added complexity imposed by data derived from a real RNA-seq experiment, we used spike-in controls that were previously added to human monocyte-derived macrophages from five different donors (Hoss *et al.*, 2019). The Spike-In RNA Variants (SIRV) (Paul *et al.*, 2016) comprise 69 synthetic RNA molecules that were added in known relative concentrations before library preparation. Mimicking the complexity of 7 human model genes, between 6 and 18 artificial transcripts per gene vary in different types of alternative splicing, transcription start- and end-sites, or are transcribed from overlapping genes, or the antisense strand. The concentration ratios between different SIRV isoforms span a range of more than two orders of magnitude. For each donor sample, including artificial SIRV isoforms, Hoss *et al.* (2019) sequenced 200 million paired-end reads of 2 × 125 bp length. McSplicer considers both mates independently as input reads *R_n_* (see Section 2.3).

**Fig. 6.**
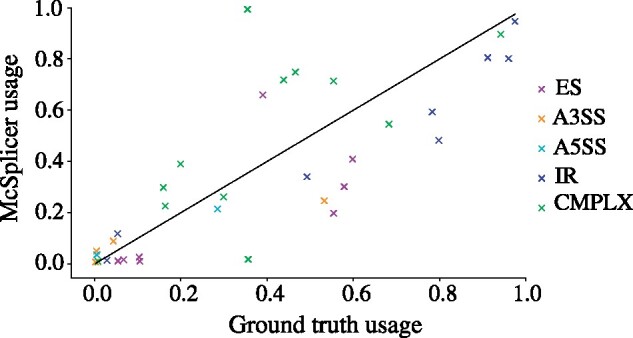
McSplicer results on spike-in RNA variants (SIRV), donor sample 5. Ground truth splice site usages computed from known mixing ratios of SIRV isoforms are compared to usages estimated by McSplicer. Out of 38 variable splice sites, 26 belong to simple events and 12 belong to complex events. ES denotes exon skipping, A3SS alternative 3′ splice site, A5SS alternative 5′ splice site, IR intron retention and CMPLX complex events

Leveraging the artificial reference genome (SIRVome) and the known relative mixing ratios of SIRV isoforms, we derive ground truth splice site usages (see Supplementary Section S2.6.3). Again, we obtain event labels from Astalavista, which comprise 26 variable splice sites in simple events and 12 in complex events. In this experiment, we do not restrict the evaluation to comparable splice sites but include all variable sites since competing methods report too few events to be compared quantitatively (see below). Figure 6 compares splice site usages as estimated by McSplicer to the true usages in one of the five samples (donor 5). A Spearman’s rank correlation coefficient of ρ=0.798 indicates a good agreement between estimated and true usages. We obtain similar results on the remaining four samples (Supplementary Fig. S13).

SplAdder and MAJIQ only report between 6 and 12 among all 38 true events, too few to allow for a meaningful quantification of agreement between estimated and true PSI and *ψ* values. Supplementary Figures S14 and S15 show the corresponding scatter plots for PSI and *ψ* values estimated by SplAdder and MAJIQ, respectively. PSGInfer failed to run on all five donor samples for unknown reasons.

### 3.4 Quantifying the effect of cryptic splice site mutations in patients with autism spectrum disorder

In this section, we illustrate the utility of splice site usages computed by McSplicer in interpreting the potentially complex effect of genetic variants on RNA splicing. In Jaganathan *et al.* (2019), the authors use a deep neural network to identify non-coding genetic variants that disrupt mRNA splicing. They identified a set of high-confidence *de novo* mutations predicted to disrupt splicing in individuals with intellectual disability and individuals with autism spectrum disorders (ASD). To validate them, the study included RNA-seq experiments (270–388 million 150 bp reads per sample) of peripheral blood-derived lymphoblastoid cell lines from 36 individuals with ASD. Based on the presence of reads spanning the corresponding splice junction, the authors validate 21 aberrant splicing events associated with the predicted *de novo* mutations. Each of the splicing events was uniquely observed in one individual.

**Fig. 7.**
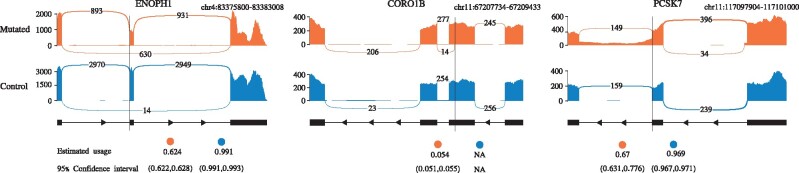
McSplicer splice site usage estimates and 95% bootstrapping confidence intervals for three disrupted splicing events reported in ASD patients versus control individuals. Variant locations are indicated by black vertical lines. Each plot illustrates the gene structure around the event with the precise genomic window specified on top, the read coverage and the junction read count. The Sashimi plots shown here are created using the ggsashimi tool (Garrido-Martín *et al.*, 2018)

In Jaganathan *et al.* (2019), the authors point out that computing the effects size of splicing mutations based on a pre-selected set of incident splice junctions likely underestimates the true effect size since, among other shortcomings, not all isoform changes are taken into account. In contrast, McSplicer’s model of splice site usage does not depend on an ad hoc selection of specific junctions or AS units but naturally captures simultaneous changes in expression of multiple isoforms expressed by a gene. We therefore utilized McSplicer to quantify the effect size of the validated *de novo* mutations on splice sites in ASD patients. We excluded 11 aberrant splicing events where only 1 or 2 spliced reads supported the novel splice site or junction. For each *de novo* mutation and the corresponding aberrant splicing event, we used McSplicer to estimate splice site usage and to compute 95% bootstrapping confidence intervals for the individual harboring the variant and a control individual with similar sequencing depth. For all 10 aberrant splicing events, we observe significantly different splice site usages (i.e. the two confidence intervals do not overlap) between mutated and control ASD individuals (Supplementary Table S3). Figure 7 provides three illustrative examples. For gene *ENOPH1*, McSplicer estimates a decrease in usage of the acceptor site directly affected by the variant, consistent with the increased skipping of the corresponding exon that can be observed in the Sashimi plot. In gene *CORO1B*, a novel donor site is used exclusively in the individual with the variant, identified and quantified with non-zero usage by McSplicer. For gene *PCSK7*, McSplicer estimates a decrease in usage of the affected donor sites, consistent with the retention of the downstream intron.

## 4 Conclusion

We have introduced McSplicer, a novel method that estimates the usage of exon start and end sites, and in particular the usage of splice sites across expressed transcripts. Rather than attempting to reconstruct expressed transcripts, McSplicer is based on a simplified probabilistic splicing model that has generated the set of expressed transcripts. It is not restricted to a pre-defined class of alternative splicing events or units but our probabilistic model is able to describe arbitrarily complex types of splicing patterns based on few, easy to interpret, parameters. We estimate these parameters, i.e. splice site usages, using all read data at once and demonstrate in simulation experiments that this yields more accurate estimates compared to other methods that use only reads directly supporting their parameters. Through its integration with transcript assembly methods such as StringTie, McSplicer quantifies the usage of annotated as well as novel splice sites.

Our model for relative transcript abundance assumes the Markovian property across indicators (*Z_i_*) for whether a segment is transcribed. This assumption allows for an efficient algorithm to estimate parameters of the model, but it potentially limits the ability of our method to model longer range dependencies such as between the recognition of 5′ and 3′ splice sites or between the removal of introns within transcripts. If true dependencies are longer than our model can describe, the individual estimators for splice site usages may still be accurate, but we expect transcript frequencies implied by our model to be less accurate (LeGault and Dewey, 2013). One way to model longer range dependencies is to use higher order Markov chains as long as the data provide sufficient information to estimate these dependencies.

The splice site usages computed by McSplicer can be leveraged in various types of downstream analyses, such as the statistical comparison of splice site usage between different conditions (Li *et al.*, 2018), the quantification of various types of splicing events, the identification of subgroups of samples that show similar splicing patterns [i.e. unsupervised clustering (Ntranos *et al.*, 2016)], or the discrimination between alternatively spliced and constitutive exons (Patrick *et al.*, 2013).

We have used McSplicer to quantify the effect size of splicing mutations in ASD patients. In this context, splice site usage as computed by McSplicer can be considered analogous to the ‘strength’ of a splice site predicted by methods such as SplicePort (Dogan *et al.*, 2007) from sequence-based features. Point mutations in the consensus splice site sequence can affect the strength of a splice site and result in the skipping of the exon or the activation of cryptic splice sites. In fact, a single nucleotide substitution might produce multiple (erroneous) splicing isoforms at the same time, as has been observed, for example, for specific mutations in patients with cystic fibrosis (3 isoforms) (Ramalho *et al.*, 2003) and X-linked spondyloepiphyseal dysplasia tarda (7 isoforms) (Xiong *et al.*, 2009). McSplicer does not attempt to reconstruct every single aberrant isoform, but similar to a weakening (strengthening) of a splice site as predicted from sequence alterations by, e.g. the Shapiro splice site probability score (Shapiro and Senapathy, 1987), the effect of a mutation will be reflected in a reduced or increased usage of the corresponding splice site estimated from RNA-seq reads.

The procedure we applied to compute the effect size of splicing mutations in our analysis of ASD patients data does not use the full data from multiple individuals and fails to consider variability among individuals, possibly leading to an increased number of false positives. Methods that model differences in splice site usages between individuals from multiple groups and exploit the variability among them should perform better in estimating effect size and quantifying their uncertainty.

## Supplementary Material

btab050_Supplementary_Data
