# Supplementary material for: McSplicer: a probabilistic model for estimating splice site usage from RNA-seq data
Source: Bioinformatics. 2021 Jan 30;37(14):2004–11. doi: 10.1093/bioinformatics/btab050 (PMC8337008; doi:10.1093/bioinformatics/btab050)
Supplement: btab050_Supplementary_Data [file btab050_supplementary_data.pdf]

Supplementary Material
McSplicer: a probabilistic model for estimating splice site usage from RNA-seq data

Israa Alqassem[1], Yash Sonthalia[2], Erika Klitzke-Feser[1], Heejung Shim[*3], and Stefan Canzar[†1]

[1]Gene Center, Ludwig-Maximilians-Universität München, Munich, 81377, Germany
[2]Google, Seattle, WA 98103, United States
[3]Melbourne Integrative Genomics (MIG), School of Mathematics and Statistics, University of Melbourne, Melbourne, 3010, Australia

1 Supplementary Figures and Tables

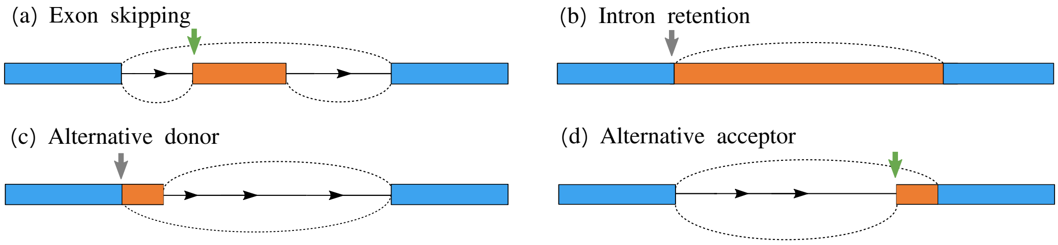

Figure S1: Four types of simple alternative splicing events. Blue rectangles represent constitutive exon or exonic segments. Orange rectangles represent alternatively spliced ones. (a) The usage of the marked acceptor site defines the relative abundance of the inclusion of the skipped exon. (b) For intron retentions, the usage of the marked splice site defines the relative abundance of the inclusion of the intron. For alternative donors (c) and alternative acceptors (d), the usage of the marked donor and acceptor sites determine the relative abundance of the two alternative events.

*heejung.shim@unimelb.edu.au
†canzar@genzentrum.lmu.de

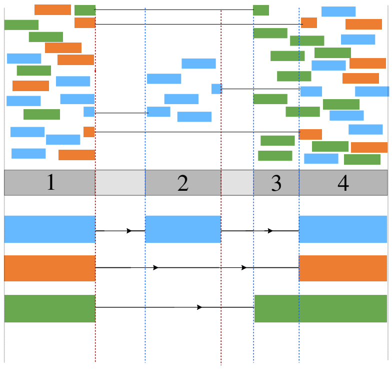

signature	signature count
{1}	16
{1 − 2}	1
{1 − 3}	1
{1 − 4}	2
{1 − 2 − 4}	0
{1 − 3 − 4}	0
{2}	4
{2 − 4}	1
{3}	4
{3 − 4}	4
{4}	15

Figure S2: An illustrative example showing signatures with their corresponding read counts. McSplicer estimates exon start and end site usages from these *signagture counts* rather than from individual read alignments. In this example, three transcripts imply a partitioning into 6 segments, 4 of which are part of exons and contain reads. Read colors indicate the originating transcript.

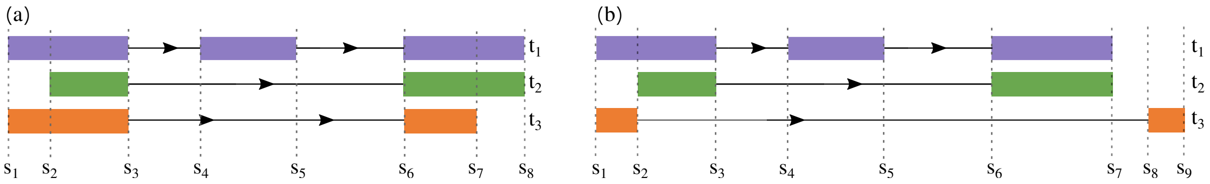

Figure S3: (a) An example of an exon skipping event involving *comparable* splice sites, see their definition in Section 2.6.2. The splice sites s_4 and s_5 are used exclusively by t_1, but splice sites s_3 and s_6 are common donor and acceptor sites to all three transcripts t_1, t_2, and t_3. (b) An example of an exon skipping event with non-comparable splice sites. The splice sites s_4 and s_5 are used exclusively by t_1. The splice sites s_3 and s_6 denote the common donor and acceptor sites of t_1 and t_2, Transcript t_3, however, is inconsistent with both t_1 and t_2 in its use of splice sites $s_3 \dots s_6$.

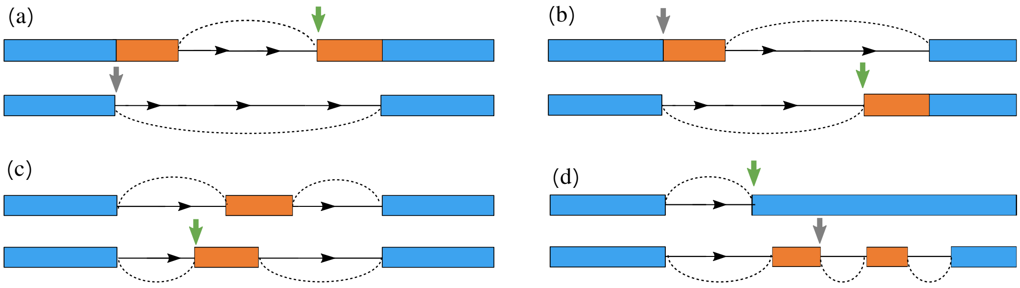

Figure S4: Examples of complex patterns of alternative splicing [2]. Green and grey arrows highlight the corresponding varying donor and acceptor splice sites, respectively. These are illustrative examples of the varying non-redundant splice sites which we consider in our benchmark.

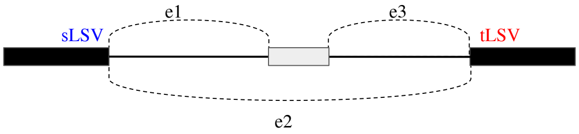

Figure S5: MAJIQ computes the percent selected index (ψ) for each junction involved in a local splicing variation (LSV) which denotes its fractional usage. An exon skipping event can be inferred either from the estimated ψ value of edge e_1 connecting source LSV ($sLSV$) to the cassette exon, or edge e_3 connecting the cassette exon to the target LSV ($tLSV$). We notice that the estimated usage $E[PSI(e_3)]$ tends to be slightly more accurate than $E[PSI(e_1)]$.

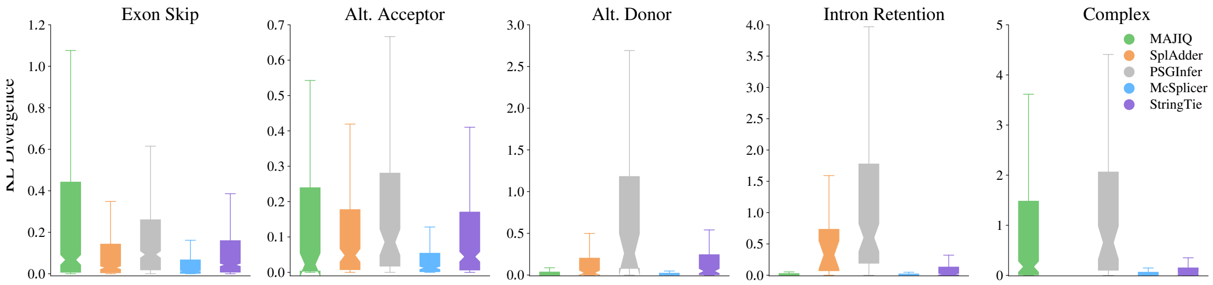

Figure S6: Accuracy of McSplicer and competing methods in quantifying the usage of variable splice sites from 50 million simulated RNA-seq reads. For MAJIQ, here we consider the estimated ψ value of the edge incident to the source LSV. See Fig. S5 for an illustration.

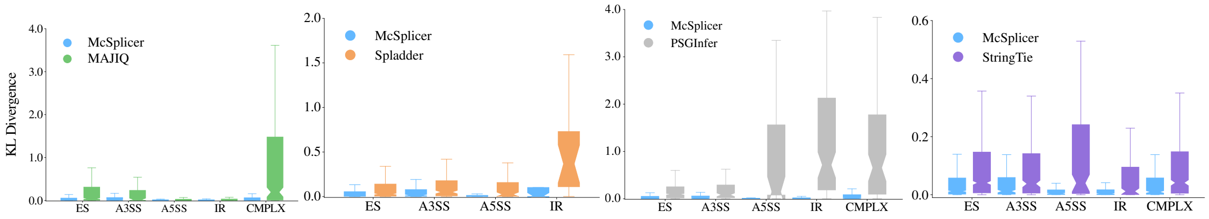

Figure S7: Accuracy of McSplicer and competing methods in quantifying the usage of variable splice sites from 50 million simulated RNA-seq reads. Events that McSplicer and competing methods have pairwise in common are considered. SplAdder is limited to the quantification of simple AS events.

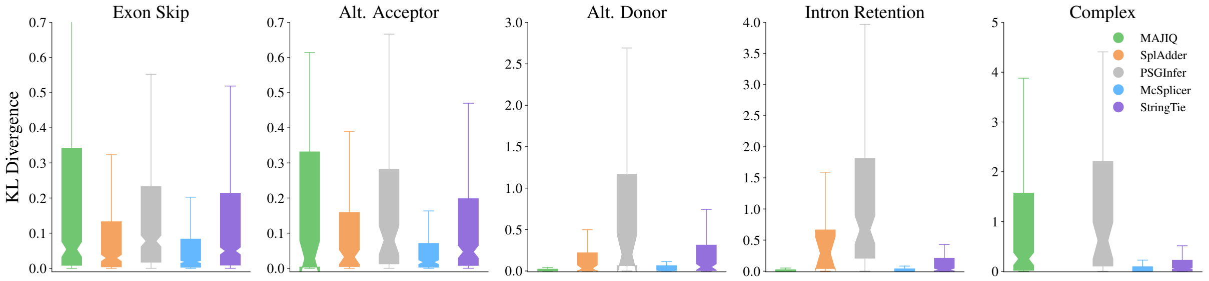

Figure S8: Accuracy of McSplicer and competing methods in quantifying the usage of variable splice sites from 20 million simulated RNA-seq reads. For each method, only splice sites in events that the method reports and quantifies are considered. SplAdder is limited to the quantification of simple events.

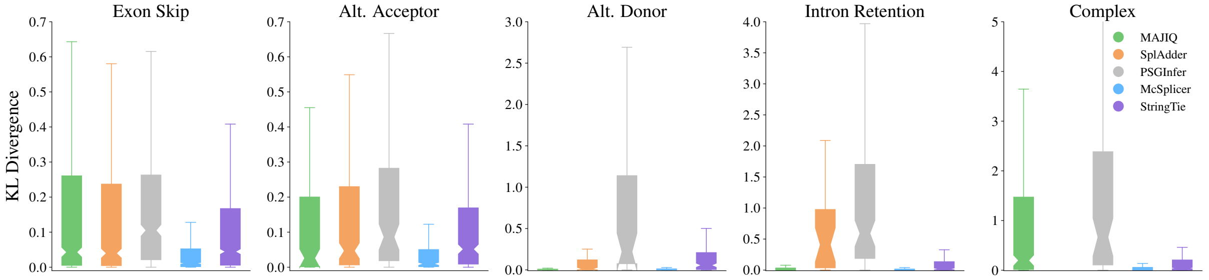

Figure S9: Accuracy of McSplicer and competing methods in quantifying the usage of variable splice sites from 75 million simulated RNA-seq reads. For each method, only splice sites in events that the method reports and quantifies are considered. SplAdder is limited to the quantification of simple events.

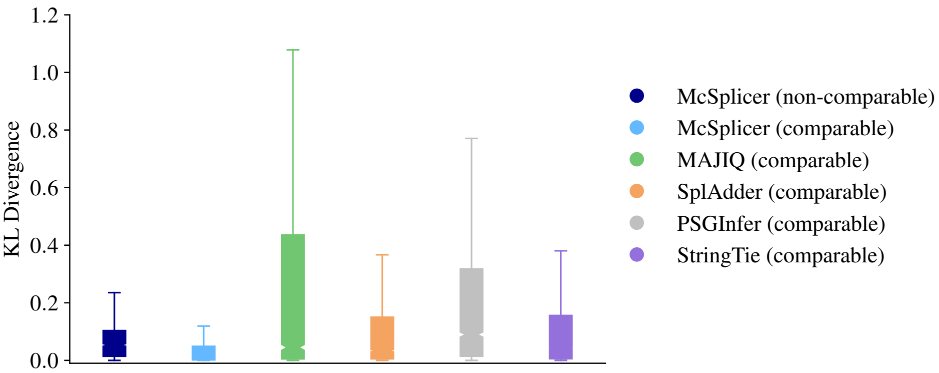

Figure S10: Accuracy of McSplicer and competing methods in quantifying the usage of comparable vs. non-comparable splice sites from 50 million simulated RNA-seq reads. For each method we report KL divergences on comparable splice sites over all types of events. For McSplicer we additionally show KL divergences for all non-comparable splice sites. Note that KL divergences reported for SplAdder do not include complex events, on which MAJIQ and PSGInfer obtained substantially less accurate estimates, see Figure 4 and Table S2. In contrast to comparable splice sites that are included or excluded in only one unique way across all expressed transcripts, non-comparable splice sites may be overlapped by an arbitrary number of transcripts with varying exon-intron structure.

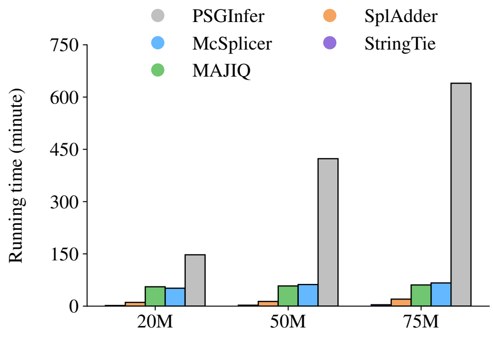

	20M	50M	75M
PSGInfer	147.37	423.17	639.72
McSplicer	51.47	62.11	66.63
MAJIQ	55.62	58.02	60.87
SplAdder	10.77	13.50	20.14
StringTie	1.93	2.77	4.06

Figure S11: Running times in minutes of PSGInfer, McSplicer, MAJIQ, SplAdder and StringTie on the 20, 50, and 75 million simulated RNA-seq reads data sets. The running time reported for McSplicer includes the time needed to partition genes into non-overlapping segments and to count reads that map to the same sequence of segments (*signature counts*, see Methods) The running times were measured on an Intel Xeon CPU @2.30GHz with 320 GB memory. McSplicer, MAJIQ, and SplAdder were run in single-thread mode, while PSGInfer was run with 72 threads to speed up computation.

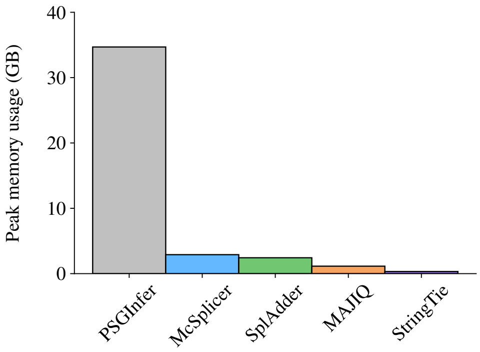

Figure S12: Peak memory usage measured for all methods on the largest simulated RNA-seq data set with 75 million reads. Peak memory usages were 34.69 GB for PSGInfer, 2.90 GB for McSplicer, 2.43 GB for SplAdder, 1.14 GB for MAJIQ, and 0.33 GB for StringTie. Note that memory usage of PSGInfer includes the read mapping step using Bowtie [3] which could not be separated from PSGInfer's inference algorithm called by a single command *psg_infer_frequencies*. All other methods exclude read mapping.

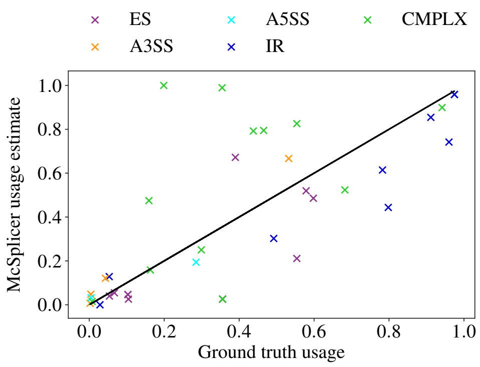

(a) Donor 1.
Spearman's $\rho = 0.774$.

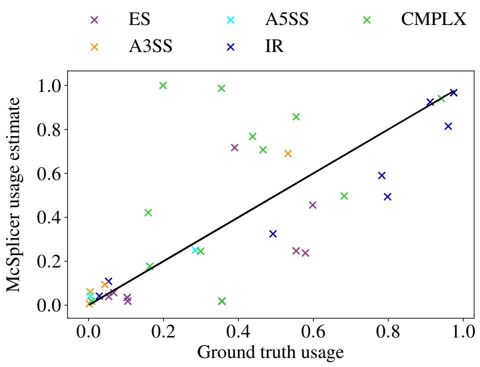

(b) Donor 2.
Spearman's $\rho = 0.769$.

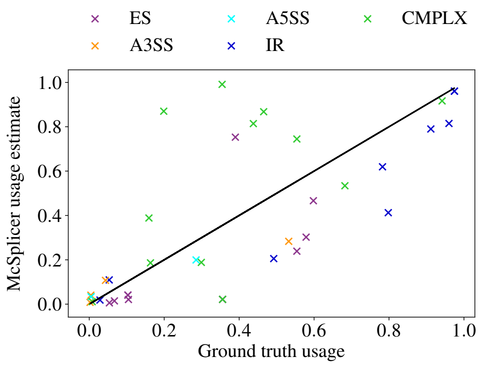

(c) Donor 3.
Spearman's $\rho = 0.774$.

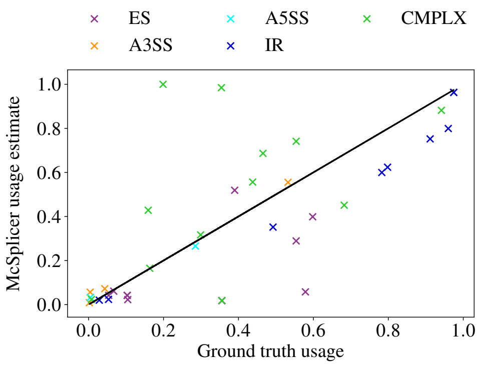

(d) Donor 4.
Spearman's $\rho = 0.782$.

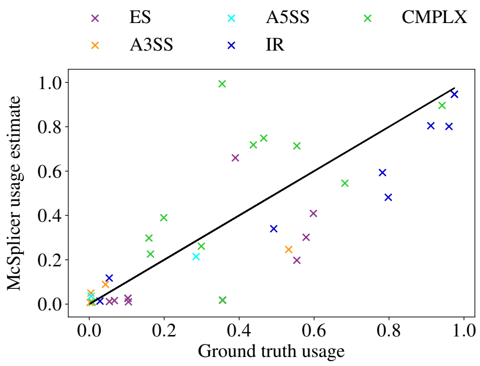

(e) Donor 5.
Spearman's $\rho = 0.798$.

Figure S13: McSplicer results on spike-in RNA variants (SIRV) on 5 different SIRV samples. Ground truth splice site usages computed from known mixing ratios of SIRV isoforms are compared to usages estimated by McSplicer. Out of 38 variable splice sites, 26 belong to simple events and 12 belong to complex events. All these variable splice sites were correctly identified by StringTie and hence their usage estimated by McSplicer. Across the five samples, StringTie reported between 1 and 6 false splice sites within SIRV genes, which corresponds to a precision in splice site detection of approximately 99%. ES: exon skipping; A5SS: alternative 5' splice site; A3SS: alternative 3' splice site; CMPLX: complex event; IR: intron retention.

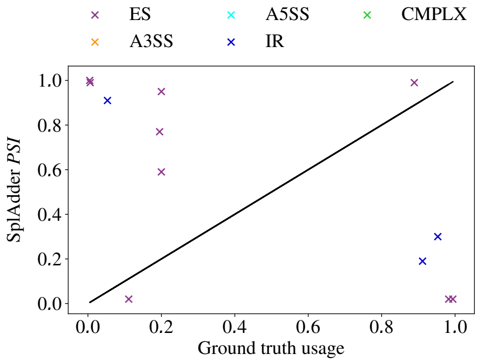

(a) Donor 1.

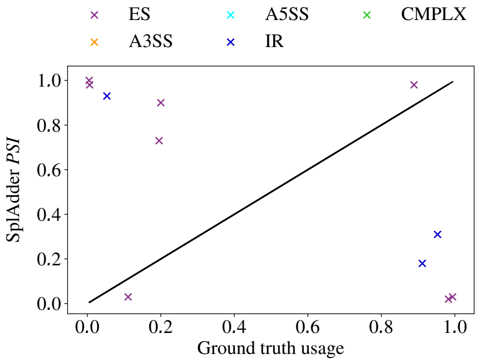

(b) Donor 2.

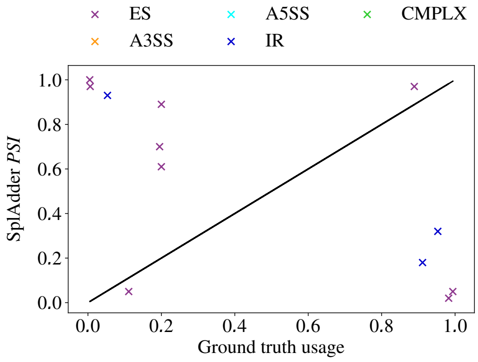

(c) Donor 3.

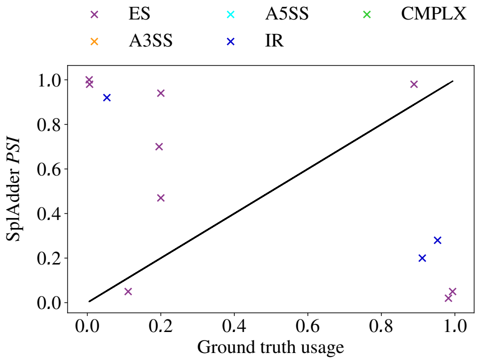

(d) Donor 4.

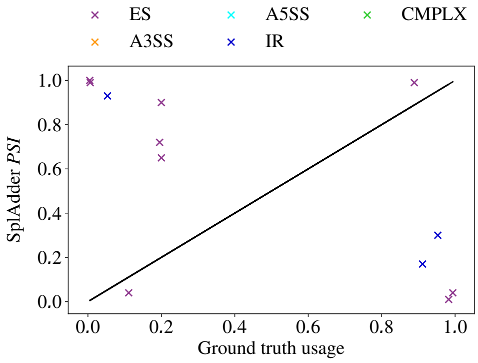

(e) Donor 5.

Figure S14: SplAdder results on spike-in RNA variants (SIRV) on 5 different SIRV samples. Ground truth splice site usages computed from known mixing ratios of SIRV isoforms are compared to usages estimated by SplAdder. Out of 38 variable splice sites, 26 belong to simple events and 12 belong to complex events. ES: exon skipping; A5SS: alternative 5' splice site; A3SS: alternative 3' splice site; CMPLX: complex event; IR: intron retention.

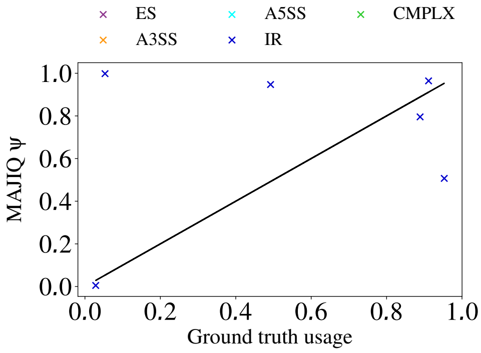

(a) Donor 1.

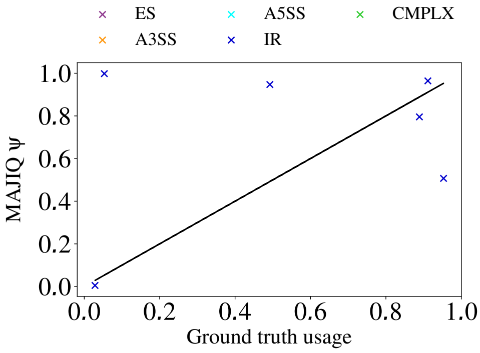

(b) Donor 2.

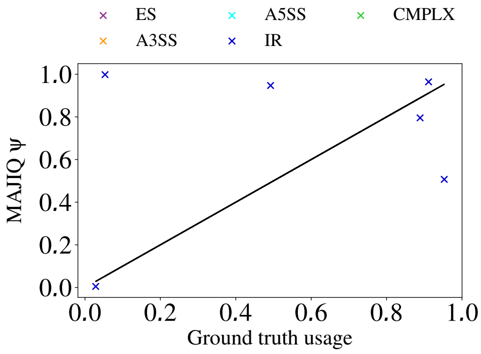

(c) Donor 3.

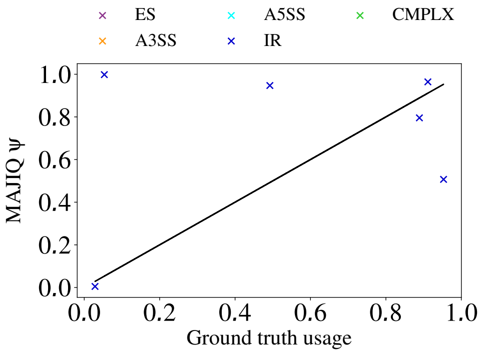

(d) Donor 4.

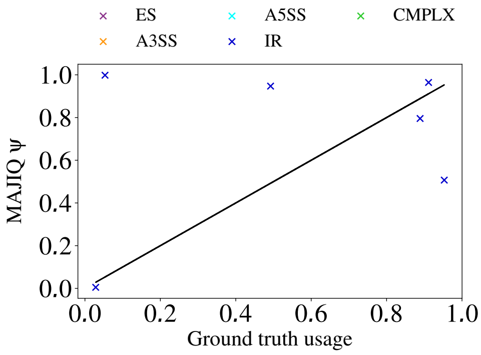

(e) Donor 5.

Figure S15: MAJIQ results on spike-in RNA variants (SIRV) on 5 different SIRV samples. Ground truth splice site usages computed from known mixing ratios of SIRV isoforms are compared to usages estimated by MAJIQ. Out of 38 variable splice sites, 26 belong to simple events and 12 belong to complex events. ES: exon skipping; A5SS: alternative 5’ splice site; A3SS: alternative 3’ splice site; CMPLX: complex event; IR: intron retention.

transcript t	$Z = (Z_1, \ldots, Z_8)$	$w(t) = \mathsf{P}(Z_1, \ldots, Z_8)$
t_1	$z_{[1:8]}(1,1,0,1,0,0,1,1)$	$\pi \times 1 \times q_1 \times p_2 \times q_2 \times (1-p_3) \times p_4 \times (1-q_3)$
t_2	$z_{[1:8]}(0,1,0,0,0,1,1,1)$	$(1-\pi) \times p_1 \times q_1 \times (1-p_2) \times 1 \times p_3 \times 1 \times (1-q_3)$
t_3	$z_{[1:8]}(1,1,0,0,0,0,1,0)$	$\pi \times 1 \times q_1 \times (1-p_2) \times 1 \times (1-p_3) \times p_4 \times q_3$

Table S1: The relative abundances defined by the McSplicer model for the three transcripts presented in Fig. 3 and Fig. S16.

	Exon skipping	Alt. acceptor	Alt. donor	Intron retention	Complex	Total
AStalavista (all)	1740	544	295	318	1206	4103
AStalavista (comparable)	475	229	129	134	508	1475
MAJIQ (comparable)	371	106	81	89	429	1076
SplAdder (comparable)	366	150	87	25	-	628
PSGInfer (comparable)	330	127	56	128	88	729
StringTie (comparable)	455	209	120	127	487	1398
McSplicer (comparable)	455	209	120	127	487	1398
McSplicer (non-comp.)	1070	292	153	180	502	2197

Table S2: The first row shows the total number of variable splice sites (i.e., comparable and non-comparable), while the second row provides the number of comparable splice sites among them. The values in the first two rows are obtained from ground truth transcript expressions and classified by type as labeled by AStalavista. Each simple event contains by definition one variable splice site whose usage uniquely quantifies the event (see Figure S1), while for complex events we consider one or two variable splice sites that are comparable (see Figure S4). The following rows show the number of variable splice sites classified by event type as quantified by each of the five methods in the simulated RNA-seq data set with 50 million reads. For McSplicer we additionally provide the number of non-comparable sites quantified. Note that McSplicer estimates the usage of all splice sites reported by StringTie. StringTie correctly identifies approximately 96% of all splice sites in our benchmark (computed from the values above) and reports few false splice sites (precision $\approx$ 96%).

Gene name	chr	Splice Site	Mutated	Control	Effect size	Event type
BCL7B	7	72966572	0.786 (0.784,0.792)	0.956 (0.953,0.960)	-1.06	ES
ENOPH1	4	83378068	0.624 (0.622, 0.628)	0.991 (0.991,0.993)	-0.20	ES
YME1L1	10	27431414	0.353 (0.349,0.356)	0.81 (0.810,0.813)	-0.36	ES
PPP4R2	3	73112824	0.463 (0.459,0.466)	0.951 (0.950,0.955)	-0.32	ES
TMBIM6	12	50153004	0.887 (0.887,0.889)	0.945 (0.948,0.949)	-0.05	ES
IDUA	4	997837	0.209 (0.208,0.211)	0.0	∞	Novel A5SS
CORO1B	11	67208804	0.054 (0.051,0.055)	0.0	∞	Novel A5SS
SHPRH	6	146266702	0.546 (0.529,0.582)	0.0	∞	Novel IR
PCSK7	11	117098932	0.67 (0.631,0.776)	0.969 (0.967,0.971)	-0.16	Novel IR
ELOVL1	1	43829994	0.200 (0.200,0.215)	0.0	∞	Novel IR

Table S3: McSplicer splice site usage estimates on mutated and control Autism samples with 95% bootstrapping confidence intervals shown in parentheses. We compute the effect size using the difference in the estimated splice site usages between mutated and control samples in log scale. There is no RNA-seq read evidence supporting the novel splice sites for the control samples in genes IDUA, CORO1B, SHPRH, and ELOVL1, hence we report the usage estimate as 0 and the effect size for these genes as ∞.

2 Methods

After introducing necessary notation in Section 2.1, we will introduce the inhomogeneous Markov chain model of McSplicer in Section 2.2, present the likelihood of the model parameters in Section 2.3, describe the EM algorithm for estimating the parameters in Section 2.4, and provide a detailed description of algorithms to compute quantities used by the EM algorithm in Section 2.5.

2.1 Notations

In this section we introduce the notation used to describe our method McSplicer. As described in the main part of this work, we assume that exon start and end sites for a gene are given. This information can be obtained from known gene annotations or inferred from RNA-seq data using methods such as StringTie. Suppose we have M_s exon start sites $s_1, \ldots, s_{M_s}$, and M_e exon end sites $e_1, \ldots, e_{M_e}$, excluding the start site of the first exon and the end site of the last exon. All exon start and end sites partition the gene into M segments, $X_1, \ldots, X_M$, where $M = M_s + M_e + 1$. We introduce a sequence of hidden variables, $Z = (Z_1, \ldots, Z_M)$, where Z_i is an indicator for whether segment X_i is part of a transcript [1] ($Z_i = 1$) or not ($Z_i = 0$).

We define a subpath s by a sequence of states for $(Z_a, \ldots, Z_b)$, $1 \le a \le b \le M$. Specifically, a subpath $s = z_{[a:b]}(o_a, \ldots, o_b)$, where $o_i \in \{0,1\}$ for $i = a, \ldots, b$, is defined by $Z_a = o_a, Z_{a+1} = o_{a+1}, \ldots, Z_b = o_b$. In other words, a subpath $s = z_{[a:b]}(o_a, \ldots, o_b)$ describes whether each of the segments from X_a to X_b belongs to a transcript or not. Then, the probability of a subpath s is:

$$\mathsf{P}(z_{[a:b]}(o_a, \ldots, o_b)) = \mathsf{P}(Z_a = o_a, Z_{a+1} = o_{a+1}, \ldots, Z_b = o_b), \tag{1}$$

which is given by our inhomogeneous Markov chain model. A path t is a subpath with $a = 1$ and $b = M$. A transcript can be represented by a path t, i.e., a sequence of states for $Z = (Z_1, \ldots, Z_M)$. Figure S16 shows an illustrative example of a gene with three transcripts which have four exon start sites and three exon end sites. These exon start and end sites divide the gene into eight segments ($M_s = 4$, $M_e = 3$, and $M = 8$). For example, a path $t = z_{[1:8]}(1,1,0,1,0,0,1,1)$ (i.e., $Z = (1,1,0,1,0,0,1,1)$) indicates transcript t_1, and a subpath $s = z_{[3:5]}(0,1,0)$ indicates a subpath obtained from the same transcript t_1.

We define the length of a subpath $s = z_{[a:b]}(o_a, \ldots, o_b)$, denoted by $l(s)$, by the number of bases included as part of a transcript:

$$l(s) = l(z_{[a:b]}(o_a, \ldots, o_b)) = \sum_{a \le i \le b : o_i = 1} l(X_i), \tag{2}$$

where $l(X_j)$ is the number of bases in segment X_j. In the example of Figure S16, let us consider a subpath of the transcript t_1, $s = z_{[3:5]}(0,1,0)$. Then, $l(s) = l(z_{[3:5]}(0,1,0)) = l(X_4)$. Similarly, we can define the length of a transcript (or a path), denoted by $l(t)$, by the number of bases included in the exonic regions of that transcript:

$$l(t) = l(z_{[1:M]}(o_1, \ldots, o_M)) = \sum_{1 \le i \le M : o_i = 1} l(X_i). \tag{3}$$

In the example shown in Figure S16, transcript t_1 has length $l(t_1) = l(z_{[1:8]}(1,1,0,1,0,0,1,1)) = l(X_1) + l(X_2) + l(X_4) + l(X_7) + l(X_8)$.

[1] We use terms transcript and isoform interchangeably to refer to a splice variant of a gene.

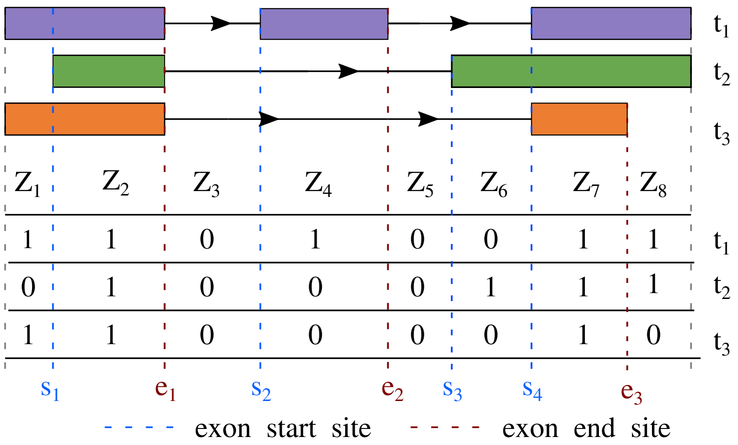

Figure S16: Hidden variables for segments defined by 3 different transcripts. The three sequences $(1,1,0,1,0,0,1,1), (0,1,0,0,0,1,1,1)$, and $(1,1,0,0,0,0,1,0)$ represent the three transcripts t_1, t_2, and t_3, respectively.

We use $F(s)$ to denote the index of the first segment in a subpath s which is part of a transcript, and use $L(s)$ to denote the index of the last segment in a subpath s which is part of a transcript. In the example shown in Figure S16, let us consider a subpath of t_1, $s = z_{[3:5]}(0,1,0)$. Then, $F(s) = 4$ and $L(s) = 4$. For transcript t_1 path $t_1 = z_{[1:8]}(1,1,0,1,0,0,1,1)$, $F(t_1) = 1$ and $L(t_1) = 8$. For transcript t_2, path $t_2 = z_{[1:8]}(0,1,0,0,0,1,1,1)$, $F(t_2) = 2$ and $L(t_2) = 8$. Similarly, for transcript t_3 path $t_3 = z_{[1:8]}(1,1,0,0,0,0,1,0)$, $F(t_3) = 1$ and $L(t_3) = 7$.

2.2 An inhomogeneous Markov chain model

In this section, we describe an inhomogeneous Markov chain to model the relative abundance of transcripts. We assume that $Z = (Z_1, \ldots, Z_M)$ follows an inhomogeneous Markov chain. Specifically, for the first segment X_1,

$$\mathsf{P}(Z_1 = 1) = \pi. \tag{4}$$

For two consecutive segments X_i and X_{i+1} for $i = 1, \ldots, M-1$ that are separated by exon start site s_m for $m = 1, \ldots, M_s$ (i.e., $i = I(s_m)$, where $I(s_m)$ is the index of the segment which appears on the left side of exon start site s_m),

$$\mathsf{P}(Z_{i+1} = 1 | Z_i = 0) = p_m, \tag{5}$$
$$\mathsf{P}(Z_{i+1} = 1 | Z_i = 1) = 1. \tag{6}$$

If they are separated by exon end site e_m for $m = 1, \ldots, M_e$ (i.e., $i = I(e_m)$, where $I(e_m)$ is the index of the segment which appears on the left side of exon end site e_m),

$$\mathsf{P}(Z_{i+1} = 0 | Z_i = 0) = 1, \tag{7}$$
$$\mathsf{P}(Z_{i+1} = 0 | Z_i = 1) = q_m. \tag{8}$$

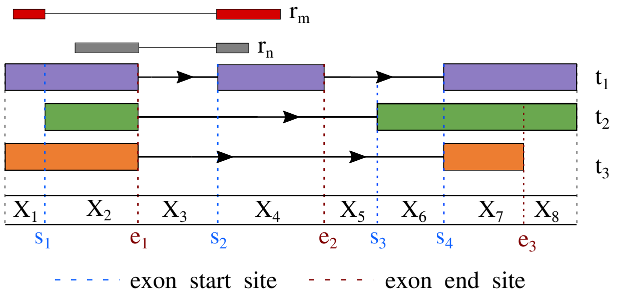

Figure S17: An example of a gene with three transcripts, the same as the one shown in Figure S16. Here, read r_n was derived from the first transcript ($T_n = t_1$) and is compatible with our model. In contrast, r_m is not compatible with our model since the two segments X_1 and X_2 are separated by exon start site s_1 and thus our model does not allow $S_m = z_{[1:4]}(1,0,0,1)$.

With this transition probability, we do not allow transcripts where $Z_i = 1$ and $Z_{i+1} = 0$ for $i = I(s_m)$, or $Z_i = 0$ and $Z_{i+1} = 1$ for $i = I(e_m)$. The parameters $p = (p_1,\ldots,p_{M_s})$ and $q = (q_1,\ldots,q_{M_e})$ indicate probabilities of using exon start sites and exon end sites, respectively. Precisely, these are conditional probabilities given that each site is considered for potential use. For example, with the current segment being part of a transcript (i.e., $Z_i = 1$), the splicing process ignores an exon start site (i.e., $\mathsf{P}(Z_{i+1} = 1|Z_i = 1) = 1$ if $i = I(s_m)$) while it considers an exon end site for potential use (i.e., $\mathsf{P}(Z_{i+1} = 0|Z_i = 1) = q_m$ if $i = I(e_m)$). Table S1 lists probabilities for the three transcripts (or paths) in Figure S16 under our Markov model. Furthermore, to handle different transcript start and end sites within a gene, we introduce artificial starting and end points (i.e., reference points) in the implementation of this model.

2.3 Likelihood of the parameters $\Theta = (\pi, p_1,\ldots,p_{M_s}, q_1,\ldots,q_{M_e})$

In this section we present the likelihood of the model parameters. Suppose we have RNA-seq reads mapped to a particular gene. The reads are derived from one end of each of the N fragments and each read has length L. We assume that each fragment is independently generated from one of the possible transcripts allowed by our model. We denote the sequence of the n-th read as r_n. T_n represents the transcript from which r_n was generated. S_n denotes the shortest subpath of T_n from which r_n is derived. B_n denotes the start position of r_n in T_n. For example, Figure S17 shows that r_n was derived from the first transcript (i.e., $T_n = t_1$), thus $T_n = z_{[1:8]}(1,1,0,1,0,0,1,1)$. The shortest subpath of T_n from which read n was derived is $S_n = z_{[2:4]}(1,0,1)$.

Assuming all r_n are derived from transcripts that are allowed in our model (i.e., $\mathsf{P}(r_n) > 0$ for all r_n), we remove reads that are not compatible with our model (see Figure S17). The likelihood of Θ can be

written as:

$$
\begin{aligned}
\mathsf{P}(r|\Theta) &= \prod_{n=1}^{N} \mathsf{P}(r_n|\Theta) \\
&= \prod_{n=1}^{N} \big[\sum_{t} \mathsf{P}(r_n, T_n = t|\Theta)\big] \\
&= \prod_{n=1}^{N} \big[\sum_{t} \big[\sum_{(s,b):s\subset t} \mathsf{P}(r_n, S_n = s, B_n = b, T_n = t|\Theta)\big]\big] \\
&\text{where } s \subset t \text{ means } s \text{ is a subpath of } t, \\
&= \prod_{n=1}^{N} \big[\sum_{t} \big[\sum_{(s,b):s\subset t} \mathsf{P}(r_n|S_n = s, B_n = b)\mathsf{P}(S_n = s, B_n = b|T_n = t)\mathsf{P}(T_n = t|\Theta)\big]\big] \qquad (9) \\
&= \prod_{n=1}^{N} \big[\sum_{t} \big[\sum_{(s,b):s\subset t,(s,b)\to r_n} 1 \frac{1}{l(t)} \frac{l(t)w_\Theta(t)}{D(\Theta)}\big]\big] \\
&\text{where } (s,b) \to r_n \text{ denotes that } r_n \text{ is the length } L \text{ sequence starting at position } b \\
&\text{in the concatenation of segments in } s, \\
&= \prod_{n=1}^{N} \big[\sum_{t} \big[\sum_{(s,b):s\subset t,(s,b)\to r_n} \frac{w_\Theta(t)}{D(\Theta)}\big]\big],
\end{aligned}
$$

where $D(\Theta) = \sum_t l(t)w_\Theta(t)$. $l(t)$ represents the (effective) length [9] of transcript t, and $w_\Theta(t)$ represents the relative frequency (probability) of transcript t.

2.4 Parameter estimation using the EM algorithm

We use an EM algorithm to compute the maximum likelihood estimate for the model parameters $\Theta = \{\pi, p, q\}$, that is, $\hat{\Theta} := \text{argmax}_\Theta \mathsf{P}(r|\Theta)$. In this section we describe the EM-steps to obtain the MLE for our model parameters. Let $Z^n = (Z_1^n, \ldots, Z_M^n)$ represent the isoform T_n, that is, the path from which read

n was derived. Then, the complete data likelihood, $\mathsf{P}(r, Z|\Theta) = \prod_{n=1}^{N} \mathsf{P}(r_n, Z^n|\Theta)$, can be written as

$$\prod_{n=1}^{N} \Big[\sum_{(s,b):s \subset Z^n} \mathsf{P}(r_n, s_n = s, b_n = b, Z^n|\Theta) \Big]$$

where $s \subset Z^n$ means s is a subpath of the path Z^n,

$$
\begin{aligned}
&= \prod_{n=1}^{N} \Big[\sum_{(s,b):s \subset Z^n} \mathsf{P}(r_n|s_n = s, b_n = b)\mathsf{P}(s_n = s, b_n = b|Z^n)\mathsf{P}(Z^n|\Theta) \Big] \\
&= \prod_{n=1}^{N} \Big[\sum_{(s,b):s \subset Z^n,(s,b) \to r_n} 1 \frac{1}{l(Z^n)} \frac{l(Z^n) w_\Theta(Z^n)}{D(\Theta)} \Big] \\
&= \prod_{n=1}^{N} \Big[\sum_{(s,b):s \subset Z^n,(s,b) \to r_n} \frac{w_\Theta(Z^n)}{D(\Theta)} \Big] \\
&= \prod_{n=1}^{N} \Big[\frac{C(r_n, Z^n) w_\Theta(Z^n)}{D(\Theta)} \Big]
\end{aligned} \tag{10}
$$

where $C(r_n, Z^n)$ indicates the number of (s, b) in the isoform T_n (defined by Z^n) which are matched to r_n. Then, we can rewrite it as

$$
\begin{aligned}
&\frac{1}{D(\Theta)^N} \prod_{n=1}^{N} \Big[C(r_n, Z^n) \big[\pi^{Z_1^n} (1-\pi)^{1-Z_1^n} \big] \\
&\times \Big[\prod_{m=1}^{M_s} p_m^{(1-Z^n_{I(s_m)})(Z^n_{I(s_m)+1})} (1-p_m)^{(1-Z^n_{I(s_m)})(1-Z^n_{I(s_m)+1})} \Big] \\
&\times \Big[\prod_{m=1}^{M_e} q_m^{(Z^n_{I(e_m)})(1-Z^n_{I(e_m)+1})} (1-q_m)^{(Z^n_{I(e_m)})(Z^n_{I(e_m)+1})} \Big] \Big].
\end{aligned} \tag{11}
$$

And we can write a log likelihood $\log \mathsf{P}(r, Z|\Theta)$ as

$$
\begin{aligned}
&-N \log D(\Theta) + \sum_{n=1}^{N} \log C(r_n, Z^n) + \sum_{n=1}^{N} Z_1^n \log \pi + \sum_{n=1}^{N} (1 - Z_1^n) \log(1-\pi) \\
&+ \sum_{n=1}^{N} \sum_{m=1}^{M_s} \Big[(1 - Z^n_{I(s_m)})(Z^n_{I(s_m)+1}) \log p_m \Big] + \sum_{n=1}^{N} \sum_{m=1}^{M_s} \Big[(1 - Z^n_{I(s_m)})(1 - Z^n_{I(s_m)+1}) \log(1-p_m) \Big] \\
&+ \sum_{n=1}^{N} \sum_{m=1}^{M_e} \Big[(Z^n_{I(e_m)})(1 - Z^n_{I(e_m)+1}) \log q_m \Big] + \sum_{n=1}^{N} \sum_{m=1}^{M_e} \Big[(Z^n_{I(e_m)})(Z^n_{I(e_m)+1}) \log(1-q_m) \Big].
\end{aligned} \tag{12}
$$

Note that the transition probabilities in our model do not allow isoforms where $Z_{I(s_m)} = 1$ and $Z_{I(s_m)+1} = 0$ at any exon start site and $Z_{I(e_m)} = 0$ and $Z_{I(e_m)+1} = 1$ at any exon end site, and $C(r_n, Z^n)$ does not depend on Θ.

2.4.1 M-step

Let $\Theta^l = (\pi^l, p_1^l, \ldots, p_{M_s}^l, q_1^l, \ldots, q_{M_e}^l)$ represent the model parameter values at the l-th iteration of the EM algorithm. Then, new parameter estimates at the $(l+1)$-th iteration are the values of Θ which maximize

$Q(\Theta \mid \Theta^l) := \mathsf{E}_{Z|r,\Theta^l}[\log \mathsf{P}(r,Z|\Theta)]$. Let $\Theta^{l+1} = (\pi^{l+1}, p_1^{l+1}, \dots, p_{M_s}^{l+1}, q_1^{l+1}, \dots, q_{M_e}^{l+1})$ denote the parameter estimates at the $(l+1)$-th iteration, then

$$\begin{aligned} \Theta^{l+1} &= \underset{\Theta}{\operatorname{argmax}}\, Q(\Theta \mid \Theta^l), \\ &= \underset{\Theta}{\operatorname{argmax}}\, \mathsf{E}_{Z|r,\Theta^l}[\log \mathsf{P}(r,Z \mid \Theta)]. \end{aligned} \tag{13}$$

We will describe how to compute Θ^{l+1} in Section 2.4.1.1 (for $p_1^{l+1}, \dots, p_{M_s}^{l+1}$), Section 2.4.1.2 (for $q_1^{l+1}, \dots, q_{M_e}^{l+1}$), and Section 2.4.1.3 (for π^{l+1}).

2.4.1.1 p_m^{l+1} for $m = 1, \dots, M_s$

Let $p'_m = 1 - p_m$ for $m = 1, \dots, M_s$, $q'_m = 1 - q_m$ for $m = 1, \dots, M_e$, and $\pi' = 1 - \pi$. Then, the Lagrangian function for maximizing $Q(\Theta \mid \Theta^l)$ is proportional to

$$\begin{aligned} \Lambda = &-N \log D(\Theta) + \sum_{n=1}^{N} \mathsf{P}(Z_1^n = 1|r_n, \Theta^l) \log \pi + \sum_{n=1}^{N} \mathsf{P}(Z_1^n = 0|r_n, \Theta^l) \log \pi' \\ &+ \sum_{n=1}^{N} \sum_{m=1}^{M_s} \Big[\mathsf{P}((1 - Z_{I(s_m)}^n)(Z_{I(s_m)+1}^n) = 1|r_n, \Theta^l) \log p_m \Big] + \sum_{n=1}^{N} \sum_{m=1}^{M_s} \Big[\mathsf{P}((1 - Z_{I(s_m)}^n)(1 - Z_{I(s_m)+1}^n) = 1|r_n, \Theta^l) \log p'_m \Big] \\ &+ \sum_{n=1}^{N} \sum_{m=1}^{M_e} \Big[\mathsf{P}((Z_{I(e_m)}^n)(1 - Z_{I(e_m)+1}^n) = 1|r_n, \Theta^l) \log q_m \Big] + \sum_{n=1}^{N} \sum_{m=1}^{M_e} \Big[\mathsf{P}((Z_{I(e_m)}^n)(Z_{I(e_m)+1}^n) = 1|r_n, \Theta^l) \log q'_m \Big] \\ &- \lambda^{\pi}(\pi + \pi' - 1) - \sum_{m=1}^{M_s} \lambda_m^s (p_m + p'_m - 1) - \sum_{m=1}^{M_e} \lambda_m^e (q_m + q'_m - 1). \end{aligned} \tag{14}$$

We take derivatives with respect to p_m and p'_m for $m = 1, \dots, M_s$ and set them to zero, leading to

$$\begin{aligned} \frac{\partial}{\partial p_m} \Lambda &= -N \frac{1}{D(\Theta)} \frac{\partial D(\Theta)}{\partial p_m} + \frac{1}{p_m} \sum_{n=1}^{N} \mathsf{P}((1 - Z_{I(s_m)}^n)(Z_{I(s_m)+1}^n) = 1|r_n, \Theta^l) - \lambda_m^s = 0, \\ \frac{\partial}{\partial p'_m} \Lambda &= -N \frac{1}{D(\Theta)} \frac{\partial D(\Theta)}{\partial p'_m} + \frac{1}{p'_m} \sum_{n=1}^{N} \mathsf{P}((1 - Z_{I(s_m)}^n)(1 - Z_{I(s_m)+1}^n) = 1|r_n, \Theta^l) - \lambda_m^s = 0. \end{aligned} \tag{15}$$

As $D(\Theta) = \sum_t l(t) w_\Theta(t) = E(l(T)) = E(l(Z))$ depends on Θ, it is difficult to find solutions for these equations. Borrowing an idea from [4], we use the fixed point iteration to solve for Θ. Thus, $\lambda_m^s = 0$ for $m = 1, \dots, M_s$ and the fixed point iteration uses the equation

$$p_m^{l+1} = p_m = \frac{A_m}{A_m + B_m}, \tag{16}$$

where

$$
\begin{aligned}
A_m &= \frac{\sum_{n=1}^{N} \mathsf{P}((1-Z^n_{I(s_m)})(Z^n_{I(s_m)+1}) = 1 | r_n, \Theta^l)}{E(l(Z_{[1:I(s_m)]})|Z_{I(s_m)} = 0) + E(l(Z_{[I(s_m)+1:M]})|Z_{I(s_m)+1} = 1)}, \\
B_m &= \frac{\sum_{n=1}^{N} \mathsf{P}((1-Z^n_{I(s_m)})(1-Z^n_{I(s_m)+1}) = 1 | r_n, \Theta^l)}{E(l(Z_{[1:I(s_m)]})|Z_{I(s_m)} = 0) + E(l(Z_{[I(s_m)+1:M]})|Z_{I(s_m)+1} = 0)},
\end{aligned}
\tag{17}
$$

and $Z_{[i:j]}$ for $i \le j$ denote a subpath $(Z_i, \ldots, Z_j)$.

Remark 1: p_m^{l+1} can be computed using only signature counts instead of individual reads. Let $c = (c_j)_{j=1}^J$ represent the signature counts over J signatures. Reads mapping to the same signature have the same subpath for S_n (i.e., the shortest subpath of T_n from which read n is derived). Suppose r_n and $r_{n'}$ are reads mapping to the same j-th signature and s_j represents a subpath corresponding to the j-th signature. Then,

$$
\begin{aligned}
\mathsf{P}((1-Z^n_{I(s_m)})(Z^n_{I(s_m)+1}) = 1 | r_n, \Theta^l) &= \mathsf{P}((1-Z^n_{I(s_m)})(Z^n_{I(s_m)+1}) = 1 | S_n = s_j, \Theta^l) \\
&= \mathsf{P}((1-Z^{n'}_{I(s_m)})(Z^{n'}_{I(s_m)+1}) = 1 | S_{n'} = s_j, \Theta^l) \\
&= \mathsf{P}((1-Z^{n'}_{I(s_m)})(Z^{n'}_{I(s_m)+1}) = 1 | r_{n'}, \Theta^l).
\end{aligned}
\tag{18}
$$

Instead of computing $\mathsf{P}((1-Z^n_{I(s_m)})(Z^n_{I(s_m)+1}) = 1 | r_n, \Theta^l)$ for all reads r_n, we can compute them using s_j for $j = 1, \ldots, J$. Therefore, A_m (and analogously B_m) can be computed using only signature counts.

Remark 2: In the E-step (Section 2.4.2) we compute $\mathsf{P}((1-Z^n_{I(s_m)})(Z^n_{I(s_m)+1}) = 1 | S_n = s_j, \Theta^l)$ and $\mathsf{P}((1-Z^n_{I(s_m)})(1-Z^n_{I(s_m)+1}) = 1 | S_n = s_j, \Theta^l)$ for $j = 1, \ldots, J$.

Remark 3: Sections 2.5.1 and 2.5.2 provide more detailed explanations of quantities $E(l(Z_{[1:I(s_m)]})|Z_{I(s_m)} = 0)$, $E(l(Z_{[I(s_m)+1:M]})|Z_{I(s_m)+1} = 1)$, $E(l(Z_{[1:I(s_m)]})|Z_{I(s_m)} = 0)$, and $E(l(Z_{[I(s_m)+1:M]})|Z_{I(s_m)+1} = 0)$, and describe how to compute them using dynamic programming.

2.4.1.2 q_m^{l+1} for $m = 1, \ldots, M_e$

Using a derivation similar to one for p_m^{l+1} above, we can obtain the following result. Let

$$
C_m = \frac{\sum_{n=1}^{N} \mathsf{P}((Z^n_{I(e_m)})(1-Z^n_{I(e_m)+1}) = 1 | r_n, \Theta^l)}{E(l(Z_{[1:I(e_m)]})|Z_{I(e_m)} = 1) + E(l(Z_{[I(e_m)+1:M]})|Z_{I(e_m)+1} = 0)}, \tag{19}
$$

$$
D_m = \frac{\sum_{n=1}^{N} \mathsf{P}((Z^n_{I(e_m)})(Z^n_{I(e_m)+1}) = 1 | r_n, \Theta^l)}{E(l(Z_{[1:I(e_m)]})|Z_{I(e_m)} = 1) + E(l(Z_{[I(e_m)+1:M]})|Z_{I(e_m)+1} = 1)}. \tag{20}
$$

Then

$$
q_m^{l+1} = \frac{C_m}{C_m + D_m}. \tag{21}
$$

Remark 1: Using a derivation similar to the one for p_m^{l+1} above, we can show that q_m^{l+1} can be computed using only signature counts.

Remark 2: In the E-step (Section 2.4.2) we compute $\mathsf{P}((Z^n_{I(e_m)})(1-Z^n_{I(e_m)+1}) = 1|S_n = s_j, \Theta^l)$ and $\mathsf{P}((Z^n_{I(e_m)})(Z^n_{I(e_m)+1}) = 1|S_n = s_j, \Theta^l)$.

Remark 3: Sections 2.5.1 and 2.5.2 provide more detailed explanations of quantities $E(l(Z_{[1:I(e_m)]})|Z_{I(e_m)} = 1)$, $E(l(Z_{[I(e_m)+1:M]})|Z_{I(e_m)+1} = 0)$, $E(l(Z_{[1:I(e_m)]})|Z_{I(e_m)} = 1)$, and $E(l(Z_{[I(e_m)+1:M]})|Z_{I(e_m)+1} = 1)$, and describe how to compute them using dynamic programming.

2.4.1.3 π^{l+1}

Using a derivation similar to one for p_m^{l+1} above, we can obtain the following result. Let

$$E = \frac{\sum_{n=1}^{N} \mathsf{P}(Z_1^n = 1|r_n, \Theta^l)}{E(l(Z_{[1:M]})|Z_1 = 1)}, \tag{22}$$

$$F = \frac{\sum_{n=1}^{N} \mathsf{P}(Z_1^n = 0|r_n, \Theta^l)}{E(l(Z_{[1:M]})|Z_1 = 0)}. \tag{23}$$

Then

$$\pi^{l+1} = \frac{E}{E+F}. \tag{24}$$

Remark 1: Using a derivation similar to the one for p_m^{l+1} above, we can show that π^{l+1} can be computed using only signature counts.

Remark 2: In the E-step (Section 2.4.2) we compute $\mathsf{P}(Z_1^n = 1|S_n = s_j, \Theta^l)$ and $\mathsf{P}(Z_1^n = 0|S_n = s_j, \Theta^l)$.

Remark 3: Section 2.5.2 provides more detailed explanations of quantities $E(l(Z_{[1:M]})|Z_1 = 1)$ and $E(l(Z_{[1:M]})|Z_1 = 0)$, and describes how to compute them using dynamic programming.

2.4.2 E-step

Let $c = (c_j)_{j=1}^J$ represent the signature counts over J signatures and s_j represents a subpath corresponding to the j-th signature.

$$\begin{aligned} \mathsf{P}((1-Z^n_{I(s_m)})(Z^n_{I(s_m)+1}) = 1|S_n = s_j, \Theta^l) &= \frac{\mathsf{P}((1-Z^n_{I(s_m)})(Z^n_{I(s_m)+1}) = 1, S_n = s_j|\Theta^l)}{\mathsf{P}(S_n = s_j|\Theta^l)}, \\ &= \frac{\mathsf{P}(Z^n_{I(s_m)} = 0, Z^n_{I(s_m)+1} = 1, S_n = s_j|\Theta^l)}{\mathsf{P}(S_n = s_j|\Theta^l)}, \end{aligned} \tag{25}$$

$$\mathsf{P}((1-Z^n_{I(s_m)})(1-Z^n_{I(s_m)+1})=1|S_n=s_j,\Theta^l)=\frac{\mathsf{P}((1-Z^n_{I(s_m)})(1-Z^n_{I(s_m)+1})=1,S_n=s_j|\Theta^l)}{\mathsf{P}(S_n=s_j|\Theta^l)},$$
$$=\frac{\mathsf{P}(Z^n_{I(s_m)}=0,Z^n_{I(s_m)+1}=0,S_n=s_j|\Theta^l)}{\mathsf{P}(S_n=s_j|\Theta^l)}, \tag{26}$$

$$\mathsf{P}((Z^n_{I(e_m)})(Z^n_{I(e_m)+1})=1|S_n=s_j,\Theta^l)=\frac{\mathsf{P}((Z^n_{I(e_m)})(Z^n_{I(e_m)+1})=1,S_n=s_j|\Theta^l)}{\mathsf{P}(S_n=s_j|\Theta^l)},$$
$$=\frac{\mathsf{P}(Z^n_{I(e_m)}=1,Z^n_{I(e_m)+1}=1,S_n=s_j|\Theta^l)}{\mathsf{P}(S_n=s_j|\Theta^l)}, \tag{27}$$

$$\mathsf{P}((Z^n_{I(e_m)})(1-Z^n_{I(e_m)+1})=1|S_n=s_j,\Theta^l)=\frac{\mathsf{P}((Z^n_{I(e_m)})(1-Z^n_{I(e_m)+1})=1,S_n=s_j|\Theta^l)}{\mathsf{P}(S_n=s_j|\Theta^l)},$$
$$=\frac{\mathsf{P}(Z^n_{I(e_m)}=1,Z^n_{I(e_m)+1}=0,S_n=s_j|\Theta^l)}{\mathsf{P}(S_n=s_j|\Theta^l)}, \tag{28}$$

$$\mathsf{P}(Z^n_1=1|S_n=s_j,\Theta^l)=\frac{\mathsf{P}(Z^n_1=1,S_n=s_j|\Theta^l)}{\mathsf{P}(S_n=s_j|\Theta^l)}, \tag{29}$$

$$\mathsf{P}(Z^n_1=0|S_n=s_j,\Theta^l)=\frac{\mathsf{P}(Z^n_1=0,S_n=s_j|\Theta^l)}{\mathsf{P}(S_n=s_j|\Theta^l)}, \tag{30}$$

where

$$\begin{aligned}&\mathsf{P}(S_n=s_j|\Theta^l)=\mathsf{P}(Z^n_1=0,S_n=s_j|\Theta^l)+\mathsf{P}(Z^n_1=1,S_n=s_j|\Theta^l)\\&=\mathsf{P}(Z^n_1=0)\mathsf{P}(Z^n_{F(s_j)}=1|Z^n_1=0)\mathsf{P}(S_n=s_j|Z^n_{F(s_j)}=1)+\mathsf{P}(Z^n_1=1)\mathsf{P}(Z^n_{F(s_j)}=1|Z^n_1=1)\mathsf{P}(S_n=s_j|Z^n_{F(s_j)}=1)\\&=(1-\pi)\mathsf{P}(Z^n_{F(s_j)}=1|Z^n_1=0)\mathsf{P}(S_n=s_j|Z^n_{F(s_j)}=1)+\pi\mathsf{P}(Z^n_{F(s_j)}=1|Z^n_1=1)\mathsf{P}(S_n=s_j|Z^n_{F(s_j)}=1).\end{aligned} \tag{31}$$

Remark 1: We describe how to compute $\mathsf{P}(S_n=s_j|Z^n_{F(s_j)}=1)$ in Section 2.5.5, $\mathsf{P}(Z^n_{I(s_m)}=0,Z^n_{I(s_m)+1}=1,S_n=s_j|\Theta^l)$ in Section 2.5.6, $\mathsf{P}(Z^n_{I(s_m)}=0,Z^n_{I(s_m)+1}=0,S_n=s_j|\Theta^l)$ in Section 2.5.7, $\mathsf{P}(Z^n_{I(e_m)}=1,Z^n_{I(e_m)+1}=1,S_n=s_j|\Theta^l)$ in Section 2.5.8, $\mathsf{P}(Z^n_{I(e_m)}=1,Z^n_{I(e_m)+1}=0,S_n=s_j|\Theta^l)$ in Section 2.5.9, and $\mathsf{P}(Z^n_1=1,S_n=s_j|\Theta^l)$ and $\mathsf{P}(Z^n_1=0,S_n=s_j|\Theta^l)$ in Section 2.5.10.

Remark 2: In Section 2.5.4 we describe the dynamic programming algorithm to compute $\mathsf{P}(Z^n_{F(s_j)}=1|Z^n_1=0)$ and $\mathsf{P}(Z^n_{F(s_j)}=1|Z^n_1=1)$.

Remark 3: $\mathsf{P}(Z^n_1=0|S_n=s_j,\Theta^l)$ can also be computed as $1-\mathsf{P}(Z^n_1=1|S_n=s_j,\Theta^l)$.

2.5 Computation of quantities used by the EM algorithm

In this section we provide a detailed description of algorithms to compute quantities used by the EM algorithm introduced in Section 2.4. Some quantities can be computed efficiently using dynamic programming (DP).

2.5.1 Expected prefix lengths: $l_p(i, \mathbf{in}) := \mathrm{E}(l(Z_{[1:i]})|Z_i = 1)$ and $l_p(i, \mathbf{out}) := \mathrm{E}(l(Z_{[1:i]})|Z_i = 0)$ for the i-th segment

The expected prefix lengths have been used in the M-step of the EM algorithm (see Section 2.4.1). In this section we formally define them and describe how to compute them using dynamic programming.

2.5.1.1 Definition

We define two types of the expected prefix length for the i-th segment, $l_p(i, \mathrm{in})$ and $l_p(i, \mathrm{out})$, as follows. Let $Z_{[1:i]}$ denote a subpath which describes a sequence of states for $(Z_1, \ldots, Z_i)$. Then, the length of the subpath $Z_{[1:i]}$ is given by

$$l(Z_{[1:i]}) = \sum_{1 \le j \le i : Z_j = 1} l(X_j), \tag{32}$$

where $l(X_j)$ indicates the number of exonic bases in the segment X_j. $l_p(i, \mathrm{in})$ is defined by the expected length of the subpath $Z_{[1:i]}$ given that X_i is a part of a transcript (i.e., $Z_i = 1$) and $l_p(i, \mathrm{out})$ is defined by the expected length of the subpath $Z_{[1:i]}$ given that X_i is not a part of a transcript (i.e., $Z_i = 0$). Specifically,

$$l_p(i, \mathrm{in}) = \mathrm{E}(l(Z_{[1:i]})|Z_i = 1) \tag{33}$$

$$l_p(i, \mathrm{out}) = \mathrm{E}(l(Z_{[1:i]})|Z_i = 0). \tag{34}$$

2.5.1.2 Computing expected prefix lengths by dynamic programming

We can compute the expected prefix lengths using dynamic programming as follows.

For $i = 1$,

$$l_p(1, \mathrm{in}) = l(X_1) \tag{35}$$

$$l_p(1, \mathrm{out}) = 0. \tag{36}$$

For $i = 2,\ldots,M$,

$$\begin{aligned}
l_p(i,\text{in}) &= \mathsf{E}(l(Z_{[1:i]})|Z_i = 1) \\
&= l(X_i) + \mathsf{E}(l(Z_{[1:(i-1)]}), Z_{i-1} = 1|Z_i = 1) + \mathsf{E}(l(Z_{[1:(i-1)]}), Z_{i-1} = 0|Z_i = 1) \\
&= l(X_i) + \mathsf{E}(l(Z_{[1:(i-1)]})|Z_{i-1} = 1, Z_i = 1)\mathsf{P}(Z_{i-1} = 1|Z_i = 1) \\
&\quad + \mathsf{E}(l(Z_{[1:(i-1)]})|Z_{i-1} = 0, Z_i = 1)\mathsf{P}(Z_{i-1} = 0|Z_i = 1) \\
&\quad \text{because } Z_{[1:(i-1)]} \text{ and } Z_i \text{ are independent conditional on } Z_{i-1} \\
&= l(X_i) + \mathsf{E}(l(Z_{[1:(i-1)]})|Z_{i-1} = 1)\mathsf{P}(Z_{i-1} = 1|Z_i = 1) \\
&\quad + \mathsf{E}(l(Z_{[1:(i-1)]})|Z_{i-1} = 0)\mathsf{P}(Z_{i-1} = 0|Z_i = 1) \\
&= l(X_i) + l_p(i-1,\text{in})\frac{\mathsf{P}(Z_{i-1} = 1)\mathsf{P}(Z_i = 1|Z_{i-1} = 1)}{\mathsf{P}(Z_i = 1)} \\
&\quad + l_p(i-1,\text{out})\frac{\mathsf{P}(Z_{i-1} = 0)\mathsf{P}(Z_i = 1|Z_{i-1} = 0)}{\mathsf{P}(Z_i = 1)}.
\end{aligned} \tag{37}$$

Similarly,

$$\begin{aligned}
l_p(i,\text{out}) &= \mathsf{E}(l(Z_{[1:i]})|Z_i = 0) \\
&= \mathsf{E}(l(Z_{[1:(i-1)]}), Z_{i-1} = 1|Z_i = 0) + \mathsf{E}(l(Z_{[1:(i-1)]}), Z_{i-1} = 0|Z_i = 0) \\
&= l_p(i-1,\text{in})\frac{\mathsf{P}(Z_{i-1} = 1)\mathsf{P}(Z_i = 0|Z_{i-1} = 1)}{\mathsf{P}(Z_i = 0)} \\
&\quad + l_p(i-1,\text{out})\frac{\mathsf{P}(Z_{i-1} = 0)\mathsf{P}(Z_i = 0|Z_{i-1} = 0)}{\mathsf{P}(Z_i = 0)}.
\end{aligned} \tag{38}$$

Remark 1: If segments X_{i-1} and X_i are separated by exon start site s_m (i.e., $i-1 = I(s_m)$),

$$\mathsf{P}(Z_i = 1|Z_{i-1} = 0) = p_m \tag{39}$$
$$\mathsf{P}(Z_i = 1|Z_{i-1} = 1) = 1, \tag{40}$$
$$\mathsf{P}(Z_i = 0|Z_{i-1} = 0) = 1 - p_m \tag{41}$$
$$\mathsf{P}(Z_i = 0|Z_{i-1} = 1) = 0, \tag{42}$$

and if segments X_{i-1} and X_i are separated by exon end site e_m (i.e., $i-1 = I(e_m)$),

$$\mathsf{P}(Z_i = 1|Z_{i-1} = 0) = 0 \tag{43}$$
$$\mathsf{P}(Z_i = 1|Z_{i-1} = 1) = 1 - q_m, \tag{44}$$
$$\mathsf{P}(Z_i = 0|Z_{i-1} = 0) = 1 \tag{45}$$
$$\mathsf{P}(Z_i = 0|Z_{i-1} = 1) = q_m. \tag{46}$$

Remark 2: Section 2.5.3 describes the dynamic programming algorithm to compute $\mathsf{P}(Z_i = 0)$ and $\mathsf{P}(Z_i = 1)$ for $i = 1,\ldots,M$.

2.5.2 Expected suffix lengths: $l_s(i,\textbf{in}) := \mathrm{E}(l(Z_{[i:M]})|Z_i = 1)$ and $l_s(i,\textbf{out}) := \mathrm{E}(l(Z_{[i:M]})|Z_i = 0)$ for the $i-th$ segment

The expected suffix lengths have been used in the M-step of the EM algorithm (see Section 2.4.1). In this section we formally define them and describe how to compute them using dynamic programming.

2.5.2.1 Definition

We define two types of expected suffix length for the $i-th$ segment, $l_s(i,\text{in})$ and $l_s(i,\text{out})$, as follows. Let $Z_{[i:M]}$ denote a subpath which describes a sequence of states for $(Z_i,\dots,Z_M)$. $l_s(i,\text{in})$ is defined by the expected length of the subpath $Z_{[i:M]}$ given that X_i is a part of an isoform (i.e., $Z_i = 1$) and $l_s(i,\text{out})$ is defined by the expected length of the subpath $Z_{[i:M]}$ given that X_i is not a part of an isoform (i.e., $Z_i = 0$). Specifically,

$$l_s(i,\text{in}) = \mathrm{E}(l(Z_{[i:M]})|Z_i = 1) \tag{47}$$

$$l_s(i,\text{out}) = \mathrm{E}(l(Z_{[i:M]})|Z_i = 0). \tag{48}$$

2.5.2.2 Computing expected suffix lengths by dynamic programming

We can compute the expected suffix lengths using dynamic programming as follows.

For $i = 1,\dots,M-1$,

$$\begin{aligned}
l_s(i,\text{in}) &= \mathrm{E}(l(Z_{[i:M]})|Z_i = 1)\\
&= l(X_i) + \mathrm{E}(l(Z_{[(i+1):M]}),Z_{i+1} = 1|Z_i = 1) + \mathrm{E}(l(Z_{[(i+1):M]}),Z_{i+1} = 0|Z_i = 1)\\
&= l(X_i) + \mathrm{E}(l(Z_{[(i+1):M]})|Z_{i+1} = 1,Z_i = 1)\mathrm{P}(Z_{i+1} = 1|Z_i = 1)\\
&+ \mathrm{E}(l(Z_{[(i+1):M]})|Z_{i+1} = 0,Z_i = 1)\mathrm{P}(Z_{i+1} = 0|Z_i = 1)\\
&\text{because } Z_{[(i+1):M]} \text{ and } Z_i \text{ are independent conditional on } Z_{i+1}\\
&= l(X_i) + \mathrm{E}(l(Z_{[(i+1):M]})|Z_{i+1} = 1)\mathrm{P}(Z_{i+1} = 1|Z_i = 1)\\
&+ \mathrm{E}(l(Z_{[(i+1):M]})|Z_{i+1} = 0)\mathrm{P}(Z_{i+1} = 0|Z_i = 1)\\
&= l(X_i) + l_s(i+1,\text{in})\mathrm{P}(Z_{i+1} = 1|Z_i = 1)\\
&+ l_s(i+1,\text{out})\mathrm{P}(Z_{i+1} = 0|Z_i = 1).
\end{aligned} \tag{49}$$

Similarly

$$\begin{aligned}
l_s(i,\text{out}) &= \mathrm{E}(l(Z_{[i:M]})|Z_i = 0)\\
&= \mathrm{E}(l(Z_{[(i+1):M]}),Z_{i+1} = 1|Z_i = 0) + \mathrm{E}(l(Z_{[(i+1):M]}),Z_{i+1} = 0|Z_i = 0)\\
&= \mathrm{E}(l(Z_{[(i+1):M]})|Z_{i+1} = 1,Z_i = 0)\mathrm{P}(Z_{i+1} = 1|Z_i = 0)\\
&+ \mathrm{E}(l(Z_{[(i+1):M]})|Z_{i+1} = 0,Z_i = 0)\mathrm{P}(Z_{i+1} = 0|Z_i = 0)\\
&= l_s(i+1,\text{in})\mathrm{P}(Z_{i+1} = 1|Z_i = 0)\\
&+ l_s(i+1,\text{out})\mathrm{P}(Z_{i+1} = 0|Z_i = 0).
\end{aligned} \tag{50}$$

And for $i = M$,

$$l_s(M, \text{in}) = l(X_M) \tag{51}$$
$$l_s(M, \text{out}) = 0. \tag{52}$$

Remark 1: For the computation of $\mathsf{P}(Z_i = 1|Z_{i-1} = 0)$, $\mathsf{P}(Z_i = 1|Z_{i-1} = 1)$, $\mathsf{P}(Z_i = 0|Z_{i-1} = 0)$, and $\mathsf{P}(Z_i = 0|Z_{i-1} = 1)$, see Remark 1 in Section 2.5.1.2.

2.5.3 Computing $\mathsf{P}(Z_i = 1)$ and $\mathsf{P}(Z_i = 0)$ using dynamic programming

The probability that segment X_i is part of a transcript, $\mathsf{P}(Z_i = 1)$, and the probability that segment X_i is not part of a transcript, $\mathsf{P}(Z_i = 0)$, have been used in the dynamic program to compute the expected prefix lengths in Section 2.5.1.2. We can compute $\mathsf{P}(Z_i = 1)$ and $\mathsf{P}(Z_i = 0)$ using dynamic programming as follows.

For $i = 1$,

$$\mathsf{P}(Z_1 = 0) = 1 - \pi, \tag{53}$$
$$\mathsf{P}(Z_1 = 1) = \pi. \tag{54}$$

For $i = 2, \ldots, M$,

$$\mathsf{P}(Z_i = 1) = \mathsf{P}(Z_{i-1} = 1)\mathsf{P}(Z_i = 1|Z_{i-1} = 1) + \mathsf{P}(Z_{i-1} = 0)\mathsf{P}(Z_i = 1|Z_{i-1} = 0), \tag{55}$$

$$\begin{aligned}\mathsf{P}(Z_i = 0) &= 1 - \mathsf{P}(Z_i = 1), \text{ or equivalently} \\ &= \mathsf{P}(Z_{i-1} = 1)\mathsf{P}(Z_i = 0|Z_{i-1} = 1) + \mathsf{P}(Z_{i-1} = 0)\mathsf{P}(Z_i = 0|Z_{i-1} = 0),\end{aligned} \tag{56}$$

Remark 1: For the computation of $\mathsf{P}(Z_i = 1|Z_{i-1} = 0)$, $\mathsf{P}(Z_i = 1|Z_{i-1} = 1)$, $\mathsf{P}(Z_i = 0|Z_{i-1} = 0)$, and $\mathsf{P}(Z_i = 0|Z_{i-1} = 1)$, see Remark 1 in Section 2.5.1.2.

2.5.4 Computing $\mathsf{P}(Z_j = 1|Z_i = 1)$, $\mathsf{P}(Z_j = 0|Z_i = 1)$, $\mathsf{P}(Z_j = 1|Z_i = 0)$, and $\mathsf{P}(Z_j = 0|Z_i = 0)$ for $1 \le i \le j \le M$ using dynamic programming

The E-step in Section 2.4.2 used $\mathsf{P}(Z_j^n = 1|Z_1^n = 0)$ and $\mathsf{P}(Z_j^n = 1|Z_1^n = 0)$ for $j = 1, \ldots, M$ to compute $\mathsf{P}(S_n = s_j|\Theta^l)$. We also use $\mathsf{P}(Z_j^n = 1|Z_i^n = 1)$, $\mathsf{P}(Z_j^n = 0|Z_i^n = 1)$, $\mathsf{P}(Z_j^n = 1|Z_i^n = 0)$, and $\mathsf{P}(Z_j^n = 0|Z_i^n = 0)$ for $1 \le i \le j \le M$ in Sections 2.5.6, 2.5.7, 2.5.8, 2.5.9, and 2.5.10. Here, we describe their computation using dynamic programming (DP). As these quantities are identical for all reads r_n, we drop superscript n in this section for simplicity.

First, let us denote the probability of Z_j conditional on Z_i as follows. For $1 \le i \le j \le M$,

$$f_{11}(i,j) := \mathsf{P}(Z_j = 1 | Z_i = 1) \quad (57)$$
$$f_{10}(i,j) := \mathsf{P}(Z_j = 0 | Z_i = 1) \quad (58)$$
$$f_{01}(i,j) := \mathsf{P}(Z_j = 1 | Z_i = 0) \quad (59)$$
$$f_{00}(i,j) := \mathsf{P}(Z_j = 0 | Z_i = 0). \quad (60)$$

We can compute these quantities using DP as follows.

2.5.4.1 When $i = j$

$$f_{11}(i,j) := \mathsf{P}(Z_j = 1 | Z_i = 1) = 1 \quad (61)$$
$$f_{10}(i,j) := \mathsf{P}(Z_j = 0 | Z_i = 1) = 0 \quad (62)$$
$$f_{01}(i,j) := \mathsf{P}(Z_j = 1 | Z_i = 0) = 0 \quad (63)$$
$$f_{00}(i,j) := \mathsf{P}(Z_j = 0 | Z_i = 0) = 1. \quad (64)$$

2.5.4.2 When $i < j$

If two segments X_{j-1} and X_j are separated by an exon start site s_m (i.e., $j-1 = I(s_m)$):

$$f_{11}(i,j) := \mathsf{P}(Z_j = 1 | Z_i = 1) = \begin{cases} 1 \text{ if } i = j-1 \\ f_{11}(i,j-1) + f_{10}(i,j-1)p_m \text{ if } i < j-1, \end{cases} \quad (65)$$

because

$$\begin{aligned} &\mathsf{P}(Z_j = 1 | Z_i = 1) \\ &= \mathsf{P}(Z_j = 1, Z_{j-1} = 1 | Z_i = 1) + \mathsf{P}(Z_j = 1, Z_{j-1} = 0 | Z_i = 1) \\ &= \mathsf{P}(Z_j = 1 | Z_{j-1} = 1, Z_i = 1)\mathsf{P}(Z_{j-1} = 1 | Z_i = 1) + \mathsf{P}(Z_j = 1 | Z_{j-1} = 0, Z_i = 1)\mathsf{P}(Z_{j-1} = 0 | Z_i = 1) \\ &= \mathsf{P}(Z_j = 1 | Z_{j-1} = 1)\mathsf{P}(Z_{j-1} = 1 | Z_i = 1) + \mathsf{P}(Z_j = 1 | Z_{j-1} = 0)\mathsf{P}(Z_{j-1} = 0 | Z_i = 1) \\ &= f_{11}(i,j-1) + p_m f_{10}(i,j-1). \end{aligned} \quad (66)$$

Similarly,

$$f_{10}(i,j) := \mathsf{P}(Z_j = 0 | Z_i = 1) = \begin{cases} 0 & \text{if } i = j-1 \\ f_{10}(i,j-1)(1-p_m) & \text{if } i < j-1. \end{cases} \quad (67)$$

$$f_{01}(i,j) := \mathsf{P}(Z_j = 1 | Z_i = 0) = \begin{cases} p_m & \text{if } i = j-1 \\ f_{01}(i,j-1) + f_{00}(i,j-1)p_m & \text{if } i < j-1. \end{cases} \quad (68)$$

$$f_{00}(i,j) := \mathsf{P}(Z_j = 0 | Z_i = 0) = \begin{cases} 1-p_m & \text{if } i = j-1 \\ f_{00}(i,j-1)(1-p_m) & \text{if } i < j-1. \end{cases} \quad (69)$$

If two segments X_{j-1} and X_j are separated by an exon end site e_m (i.e., $j-1 = I(e_m)$):

$$f_{11}(i,j) := \mathsf{P}(Z_j = 1|Z_i = 1) = \begin{cases} 1-q_m & \text{if } i = j-1 \\ f_{11}(i,j-1)(1-q_m) & \text{if } i < j-1. \end{cases} \tag{70}$$

$$f_{10}(i,j) := \mathsf{P}(Z_j = 0|Z_i = 1) = \begin{cases} q_m & \text{if } i = j-1 \\ f_{10}(i,j-1) + f_{11}(i,j-1)q_m & \text{if } i < j-1. \end{cases} \tag{71}$$

$$f_{01}(i,j) := \mathsf{P}(Z_j = 1|Z_i = 0) = \begin{cases} 0 & \text{if } i = j-1 \\ f_{01}(i,j-1)(1-q_m) & \text{if } i < j-1. \end{cases} \tag{72}$$

$$f_{00}(i,j) := \mathsf{P}(Z_j = 0|Z_i = 0) = \begin{cases} 1 & \text{if } i = j-1 \\ f_{00}(i,j-1) + f_{01}(i,j-1)q_m & \text{if } i < j-1. \end{cases} \tag{73}$$

2.5.5 Computation of $w(s) = \mathsf{P}(S = s|Z_{F(s)} = 1)$

The E-step in Section 2.4.2 used $\mathsf{P}(S_n = s|Z^n_{F(s)} = 1)$ to compute $\mathsf{P}(S_n = s|\Theta^l)$. These probabilities are also used in Sections 2.5.6, 2.5.7, 2.5.8, 2.5.9, and 2.5.10. Here we describe in detail how to compute them. As these quantities are identical for all reads r_n, we drop index n in this section for simplicity.

We use $w(s)$ to denote the probability of $S = s$ conditional on $X_{F(s)}$ is a part of an isoform. Let a subpath $s = z_{[a:b]}(o_a, \ldots, o_b)$. Due to the definition of S, that is the shortest subpath of T from which a read is derived, $o_a = 1, o_b = 1, F(s) = a$ and $L(s) = b$.

$$\begin{aligned} w(s) &= \mathsf{P}(S = s|Z_{F(s)} = 1) \\ &= \frac{\mathsf{P}(S = s)}{\mathsf{P}(Z_{F(s)} = 1)} \\ &= \frac{\mathsf{P}(Z_a = o_a, Z_{a+1} = o_{a+1}, \ldots, Z_b = o_b)}{\mathsf{P}(Z_a = 1)} \\ &= \mathsf{P}(Z_{a+1} = o_{a+1}, \ldots, Z_b = o_b|Z_a = 1). \end{aligned} \tag{74}$$

Moreover,

$$\begin{aligned} w(s) &= \mathsf{P}(Z_{a+1} = o_{a+1}, \ldots, Z_b = o_b|Z_a = 1) \\ &= \prod_{i=a}^{b-1} \mathsf{P}(Z_{i+1} = o_{i+1}|Z_i = o_i) \\ &= \prod_{s_m : a \le I(s_m) < b} p_m^{(1-o_{I(s_m)})(o_{[I(s_m)+1]})} (1-p_m)^{(1-o_{I(s_m)})(1-o_{[I(s_m)+1]})} \\ &\times \prod_{e_m : a \le I(e_m) < b} q_m^{(o_{I(e_m)})(1-o_{[I(e_m)+1]})} (1-q_m)^{(o_{I(e_m)})(o_{[I(e_m)+1]})}. \end{aligned} \tag{75}$$

In the example of Figure S18, $s = z_{[2:5]}(1,1,0,1)$. So $a = 2, b = 5, o_a = 1, o_b = 1, F(s) = 2$ and $L(s) = 5$.

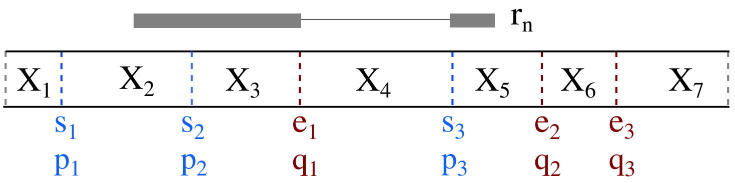

Figure S18: In this example, $s = z_{[2:5]}(1,1,0,1)$ and $w(s) = \mathsf{P}(Z_3 = 1, Z_4 = 0, Z_5 = 1 | Z_2 = 1) = 1 \cdot q_1 \cdot p_3$

Thus,

$$
\begin{aligned}
w(s) &= \mathsf{P}(Z_3 = 1, Z_4 = 0, Z_5 = 1 | Z_2 = 1) \\
&= \mathsf{P}(Z_3 = 1 | Z_2 = 1)\mathsf{P}(Z_4 = 0 | Z_3 = 1)\mathsf{P}(Z_5 = 1 | Z_4 = 0) \\
&= 1 \cdot q_1 \cdot p_3 \\
&\text{or,} \\
&= \prod_{s_m : 2 \le I(s_m) < 5} p_m^{(1-o_{I(s_m)})(o_{[I(s_m)+1]})} (1-p_m)^{(1-o_{I(s_m)})(1-o_{[I(s_m)+1]})} \\
&\times \prod_{e_m : 2 \le I(e_m) < 5} q_m^{(o_{I(e_m)})(1-o_{[I(e_m)+1]})} (1-q_m)^{(o_{I(e_m)})(o_{[I(e_m)+1]})} \\
&= \prod_{s_m : s_2, s_3} p_m^{(1-o_{I(s_m)})(o_{[I(s_m)+1]})} (1-p_m)^{(1-o_{I(s_m)})(1-o_{[I(s_m)+1]})} \\
&\times \prod_{e_m : e_1} q_m^{(o_{I(e_m)})(1-o_{[I(e_m)+1]})} (1-q_m)^{(o_{I(e_m)})(o_{[I(e_m)+1]})} \\
&= p_2^{(1-1)(1)} (1-p_2)^{(1-1)(1-1)} p_3^{(1-0)(1)} (1-p_3)^{(1-0)(1-1)} \\
&\times q_1^{(1)(1-0)} (1-q_1)^{(1)(0)} \\
&= 1 \cdot p_3 \times q_1 .
\end{aligned}
\tag{76}
$$

2.5.6 Computation of $\mathsf{P}(Z^n_{I(s_m)} = 0, Z^n_{I(s_m)+1} = 1, S_n = s)$

The E-step in Section 2.4.2 used $\mathsf{P}(Z^n_{I(s_m)} = 0, Z^n_{I(s_m)+1} = 1, S_n = s)$. Here, we describe how to compute these probabilities for the different cases when an exon start site s_m appears to the left, to the right, or within a subpath s. Figure S19 illustrates the different cases. As these quantities are identical for all reads r_n, we drop index n in this section for simplicity.

2.5.6.1 case 1: $I(s_m) < F(s)$

As shown in Figure S19, an exon start site s_m appears left side of a subpath $s = z_{[a:b]}(o_a,\ldots,o_b)$.

$$
\begin{aligned}
&\mathsf{P}(Z_{I(s_m)} = 0, Z_{I(s_m)+1} = 1, S = s) \\
&= \mathsf{P}(Z_1 = 0, Z_{I(s_m)} = 0, Z_{I(s_m)+1} = 1, S = s) + \mathsf{P}(Z_1 = 1, Z_{I(s_m)} = 0, Z_{I(s_m)+1} = 1, S = s) \\
&= \mathsf{P}(Z_1 = 0)\mathsf{P}(Z_{I(s_m)} = 0|Z_1 = 0)\mathsf{P}(Z_{I(s_m)+1} = 1|Z_{I(s_m)} = 0)\mathsf{P}(Z_{F(s)} = 1|Z_{I(s_m)+1} = 1)\mathsf{P}(S = s|Z_{F(s)} = 1) \\
&+ \mathsf{P}(Z_1 = 1)\mathsf{P}(Z_{I(s_m)} = 0|Z_1 = 1)\mathsf{P}(Z_{I(s_m)+1} = 1|Z_{I(s_m)} = 0)\mathsf{P}(Z_{F(s)} = 1|Z_{I(s_m)+1} = 1)\mathsf{P}(S = s|Z_{F(s)} = 1) \\
&= (1-\pi)f_{00}(1, I(s_m))p_m f_{11}(I(s_m)+1, F(s))w(s) + \pi f_{10}(1, I(s_m))p_m f_{11}(I(s_m)+1, F(s))w(s) \\
&= \left[(1-\pi)f_{00}(1, I(s_m)) + \pi f_{10}(1, I(s_m))\right] \times p_m f_{11}(I(s_m)+1, F(s))w(s),
\end{aligned}
\tag{77}
$$

where $f_{\cdot\cdot}(i,j)$ and $w(s)$ can be computed as described in Sections 2.5.4 and 2.5.5.

2.5.6.2 case 2: $L(s) < I(s_m)$

As shown in Figure S19, s_m appears right side of the subpath $s = z_{[a:b]}(o_a,\ldots,o_b)$.

$$
\begin{aligned}
&\mathsf{P}(S = s, Z_{I(s_m)} = 0, Z_{I(s_m)+1} = 1) \\
&= \mathsf{P}(Z_1 = 0, S = s, Z_{I(s_m)} = 0, Z_{I(s_m)+1} = 1) + \mathsf{P}(Z_1 = 1, S = s, Z_{I(s_m)} = 0, Z_{I(s_m)+1} = 1) \\
&= \mathsf{P}(Z_1 = 0)\mathsf{P}(Z_{F(s)} = 1|Z_1 = 0)\mathsf{P}(S = s|Z_{F(s)} = 1)\mathsf{P}(Z_{I(s_m)} = 0|Z_{L(s)} = 1)\mathsf{P}(Z_{I(s_m)+1} = 1|Z_{I(s_m)} = 0) \\
&+ \mathsf{P}(Z_1 = 1)\mathsf{P}(Z_{F(s)} = 1|Z_1 = 1)\mathsf{P}(S = s|Z_{F(s)} = 1)\mathsf{P}(Z_{I(s_m)} = 0|Z_{L(s)} = 1)\mathsf{P}(Z_{I(s_m)+1} = 1|Z_{I(s_m)} = 0) \\
&= (1-\pi)f_{01}(1, F(s))w(s)f_{10}(L(s), I(s_m))p_m + \pi f_{11}(1, F(s))w(s)f_{10}(L(s), I(s_m))p_m \\
&= \left[(1-\pi)f_{01}(1, F(s)) + \pi f_{11}(1, F(s))\right] \times w(s)f_{10}(L(s), I(s_m))p_m,
\end{aligned}
\tag{78}
$$

where $f_{\cdot\cdot}(i,j)$ and $w(s)$ can be computed as described in Sections 2.5.4 and 2.5.5.

2.5.6.3 case 3: $F(s) \leq I(s_m) < L(s)$ and $(Z_{I(s_m)} = 0, Z_{I(s_m)+1} = 1)$ is a subset of s

A subpath $s = z_{[a:b]}(o_a,\ldots,o_b)$ can be represented by $(Z_a = o_a, Z_{a+1} = o_{a+1},\ldots,Z_b = o_b)$. If the subpath s contains $(Z_{I(s_m)} = 0, Z_{I(s_m)+1} = 1)$, then $(Z_{I(s_m)} = 0, Z_{I(s_m)+1} = 1)$ is a subset of s (i.e., $(Z_{I(s_m)} = 0, Z_{I(s_m)+1} = 1) \subset s$). As shown in Figure S19, s_m appears inside of the subpath $s = z_{[a:b]}(o_a,\ldots,o_b)$.

$$
\begin{aligned}
&\mathsf{P}(S = s, Z_{I(s_m)} = 0, Z_{I(s_m)+1} = 1) \\
&= \mathsf{P}(S = s) \\
&= \mathsf{P}(Z_1 = 0, S = s) + \mathsf{P}(Z_1 = 1, S = s) \\
&= \mathsf{P}(Z_1 = 0)\mathsf{P}(Z_{F(s)} = 1|Z_1 = 0)\mathsf{P}(S = s|Z_{F(s)} = 1) + \mathsf{P}(Z_1 = 1)\mathsf{P}(Z_{F(s)} = 1|Z_1 = 1)\mathsf{P}(S = s|Z_{F(s)} = 1) \\
&= (1-\pi)f_{01}(1, F(s))w(s) + \pi f_{11}(1, F(s))w(s) \\
&= \left[(1-\pi)f_{01}(1, F(s)) + \pi f_{11}(1, F(s))\right] \times w(s),
\end{aligned}
\tag{79}
$$

where $f_{\cdot\cdot}(i,j)$ and $w(s)$ can be computed as described in Sections 2.5.4 and 2.5.5.

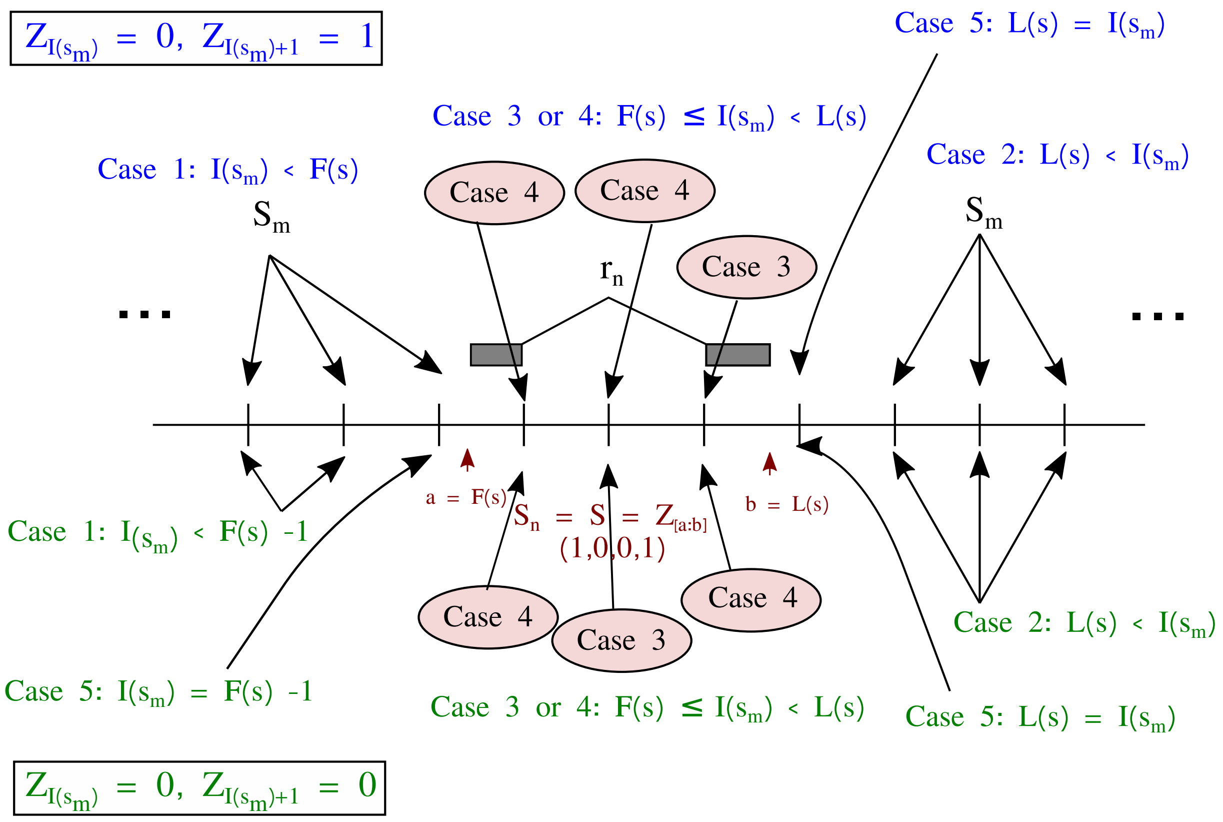

Figure S19: Visualization of the different cases considered for computing $\mathsf{P}(Z^n_{I(s_m)} = 0, Z^n_{I(s_m)+1} = 1, S_n = s)$ in the upper part, and $\mathsf{P}(Z^n_{I(s_m)} = 0, Z^n_{I(s_m)+1} = 0, S_n = s)$ in the lower part. Arrows from each case point to an exon start site or a set of exon start sites. a and b represent the indices of the first and last segments of the subpath $S_n = s$ from which read r_n is derived. In the upper part for $\mathsf{P}(Z^n_{I(s_m)} = 0, Z^n_{I(s_m)+1} = 1, S_n = s)$, an exon start site s_m appears to the left of subpath s (case 1), to the right of s (cases 2 and 5), or within s (cases 3 and 4). We do not allow for cases 4 and 5 where $Z^n_{I(s_m)} = 0$ and $Z^n_{I(s_m)+1} = 1$ are not compatible with subpath s. In the lower part for $\mathsf{P}(Z^n_{I(s_m)} = 0, Z^n_{I(s_m)+1} = 0, S_n = s)$, s_m appears to the left of s (cases 1 and 5), to the right of s (cases 2 and 5), or within s (cases 3 and 4). We do not allow for cases 4 and 5 where $Z^n_{I(s_m)} = 0$ and $Z^n_{I(s_m)+1} = 0$ are not compatible with subpath s.

2.5.6.4 case 4: $F(s) \le I(s_m) < L(s)$ and $(Z_{I(s_m)} = 0, Z_{I(s_m)+1} = 1)$ is not a subset of s

In this case, $Z_{I(s_m)} = 0$ and $Z_{I(s_m)+1} = 1$ are not compatible with subpath s.

$$\mathsf{P}(S = s, Z_{I(s_m)} = 0, Z_{I(s_m)+1} = 1) = 0 \tag{80}$$

2.5.6.5 case 5: $I(s_m) = L(s)$

In this case, $Z_{I(s_m)} = 0$ and $Z_{I(s_m)+1} = 1$ are not compatible with subpath s.

$$\mathsf{P}(S = s, Z_{I(s_m)} = 0, Z_{I(s_m)+1} = 1) = 0 \tag{81}$$

2.5.7 Computation of $\mathsf{P}(Z^n_{I(s_m)} = 0, Z^n_{I(s_m)+1} = 0, S_n = s)$

The E-step in Section 2.4.2 used $\mathsf{P}(Z^n_{I(s_m)} = 0, Z^n_{I(s_m)+1} = 0, S_n = s)$. Here, we describe how to compute these probabilities for the different cases when an exon start site s_m appears to the left, to the right, or within a subpath s. Figure S19 illustrates the different cases. As these quantities are identical for all reads r_n, we drop index n in this section for simplicity.

2.5.7.1 case 1: $I(s_m) < F(s) - 1$

As shown in Figure S19, an exon start site s_m appears left side of a subpath $s = z_{[a:b]}(o_a, \dots, o_b)$.

$$\begin{aligned}
&\mathsf{P}(Z_{I(s_m)} = 0, Z_{I(s_m)+1} = 0, S = s) \\
&= \mathsf{P}(Z_1 = 0, Z_{I(s_m)} = 0, Z_{I(s_m)+1} = 0, S = s) + \mathsf{P}(Z_1 = 1, Z_{I(s_m)} = 0, Z_{I(s_m)+1} = 0, S = s) \\
&= \mathsf{P}(Z_1 = 0)\mathsf{P}(Z_{I(s_m)} = 0|Z_1 = 0)\mathsf{P}(Z_{I(s_m)+1} = 0|Z_{I(s_m)} = 0)\mathsf{P}(Z_{F(s)} = 1|Z_{I(s_m)+1} = 0)\mathsf{P}(S = s|Z_{F(s)} = 1) \\
&+ \mathsf{P}(Z_1 = 1)\mathsf{P}(Z_{I(s_m)} = 0|Z_1 = 1)\mathsf{P}(Z_{I(s_m)+1} = 0|Z_{I(s_m)} = 0)\mathsf{P}(Z_{F(s)} = 1|Z_{I(s_m)+1} = 0)\mathsf{P}(S = s|Z_{F(s)} = 1) \\
&= (1-\pi)f_{00}(1, I(s_m))(1-p_m)f_{01}(I(s_m)+1, F(s))w(s) + \pi f_{10}(1, I(s_m))(1-p_m)f_{01}(I(s_m)+1, F(s))w(s) \\
&= \big[(1-\pi)f_{00}(1, I(s_m)) + \pi f_{10}(1, I(s_m))\big] \times (1-p_m)f_{01}(I(s_m)+1, F(s))w(s),
\end{aligned} \tag{82}$$

where $f_{\cdot\cdot}(i, j)$ and $w(s)$ can be computed as described in Sections 2.5.4 and 2.5.5.

2.5.7.2 case 2: $L(s) < I(s_m)$

As shown in Figure S19, s_m appears right side of a subpath $s = z_{[a:b]}(o_a, \dots, o_b)$.

$$\begin{aligned}
&\mathsf{P}(S = s, Z_{I(s_m)} = 0, Z_{I(s_m)+1} = 0) \\
&= \mathsf{P}(Z_1 = 0, S = s, Z_{I(s_m)} = 0, Z_{I(s_m)+1} = 0) + \mathsf{P}(Z_1 = 1, S = s, Z_{I(s_m)} = 0, Z_{I(s_m)+1} = 0) \\
&= \mathsf{P}(Z_1 = 0)\mathsf{P}(Z_{F(s)} = 1|Z_1 = 0)\mathsf{P}(S = s|Z_{F(s)} = 1)\mathsf{P}(Z_{I(s_m)} = 0|Z_{L(s)} = 1)\mathsf{P}(Z_{I(s_m)+1} = 0|Z_{I(s_m)} = 0) \\
&+ \mathsf{P}(Z_1 = 1)\mathsf{P}(Z_{F(s)} = 1|Z_1 = 1)\mathsf{P}(S = s|Z_{F(s)} = 1)\mathsf{P}(Z_{I(s_m)} = 0|Z_{L(s)} = 1)\mathsf{P}(Z_{I(s_m)+1} = 0|Z_{I(s_m)} = 0) \\
&= (1-\pi)f_{01}(1, F(s))w(s)f_{10}(L(s), I(s_m))(1-p_m) + \pi f_{11}(1, F(s))w(s)f_{10}(L(s), I(s_m))(1-p_m) \\
&= \big[(1-\pi)f_{01}(1, F(s)) + \pi f_{11}(1, F(s))\big] \times w(s)f_{10}(L(s), I(s_m))(1-p_m),
\end{aligned} \tag{83}$$

where $f_{\cdot\cdot}(i, j)$ and $w(s)$ can be computed as described in Sections 2.5.4 and 2.5.5.

2.5.7.3 case 3: $F(s) \leq I(s_m) < L(s)$ and $(Z_{I(s_m)} = 0, Z_{I(s_m)+1} = 0)$ is a subset of s

As shown in Figure S19, s_m appears inside of a subpath $s = z_{[a:b]}(o_a, \ldots, o_b)$.

$$\begin{aligned}
&\mathsf{P}(S = s, Z_{I(s_m)} = 0, Z_{I(s_m)+0} = 1) \\
&= \mathsf{P}(S = s) \\
&= \mathsf{P}(Z_1 = 0, S = s) + \mathsf{P}(Z_1 = 1, S = s) \\
&= \mathsf{P}(Z_1 = 0)\mathsf{P}(Z_{F(s)} = 1 | Z_1 = 0)\mathsf{P}(S = s | Z_{F(s)} = 1) + \mathsf{P}(Z_1 = 1)\mathsf{P}(Z_{F(s)} = 1 | Z_1 = 1)\mathsf{P}(S = s | Z_{F(s)} = 1) \\
&= (1-\pi) f_{01}(1, F(s)) w(s) + \pi f_{11}(1, F(s)) w(s) \\
&= \left[(1-\pi) f_{01}(1, F(s)) + \pi f_{11}(1, F(s))\right] \times w(s),
\end{aligned} \tag{84}$$

where $f_{..}(i, j)$ and $w(s)$ can be computed as described in Sections 2.5.4 and 2.5.5.

2.5.7.4 case 4: $F(s) \leq I(s_m) < L(s)$ and $(Z_{I(s_m)} = 0, Z_{I(s_m)+1} = 0)$ is not a subset of s

In this case, $Z_{I(s_m)} = 0$ and $Z_{I(s_m)+1} = 0$ are not compatible with subpath s.

$$\mathsf{P}(s, Z_{I(s_m)} = 0, Z_{I(s_m)+1} = 0) = 0 \tag{85}$$

2.5.7.5 case 5: $I(s_m) = F(s) - 1$ or $I(s_m) = L(s)$

In this case, $Z_{I(s_m)} = 0$ and $Z_{I(s_m)+1} = 0$ are not compatible with subpath s.

$$\mathsf{P}(s, Z_{I(s_m)} = 0, Z_{I(s_m)+1} = 0) = 0 \tag{86}$$

2.5.8 Computation of $\mathsf{P}(Z^n_{I(e_m)} = 1, Z^n_{I(e_m)+1} = 1, S_n = s)$

The E-step in Section 2.4.2 used $\mathsf{P}(Z^n_{I(e_m)} = 1, Z^n_{I(e_m)+1} = 1, S_n = s)$. Here, we describe how to compute these probabilities for the different cases when an exon end site e_m appears to the left, to the right, or within a subpath s. Figure S20 illustrates the different cases. As these quantities are identical for all reads r_n, we drop index n in this section for simplicity.

2.5.8.1 case 1: $I(e_m) < F(s)$

As shown in Figure S20, an exon end site e_m appears left side of a subpath $s = z_{[a:b]}(o_a, \ldots, o_b)$.

$$\begin{aligned}
&\mathsf{P}(Z_{I(e_m)} = 1, Z_{I(e_m)+1} = 1, S = s) \\
&= \mathsf{P}(Z_1 = 0, Z_{I(e_m)} = 1, Z_{I(e_m)+1} = 1, S = s) + \mathsf{P}(Z_1 = 1, Z_{I(e_m)} = 1, Z_{I(e_m)+1} = 1, S = s) \\
&= (1-\pi) f_{01}(1, I(e_m))(1-q_m) f_{11}(I(e_m)+1, F(s)) w(s) + \pi f_{11}(1, I(e_m))(1-q_m) f_{11}(I(e_m)+1, F(s)) w(s) \\
&= \left[(1-\pi) f_{01}(1, I(e_m)) + \pi f_{11}(1, I(e_m))\right] \times (1-q_m) f_{11}(I(e_m)+1, F(s)) w(s),
\end{aligned} \tag{87}$$

where $f_{..}(i, j)$ and $w(s)$ can be computed as described in Sections 2.5.4 and 2.5.5.

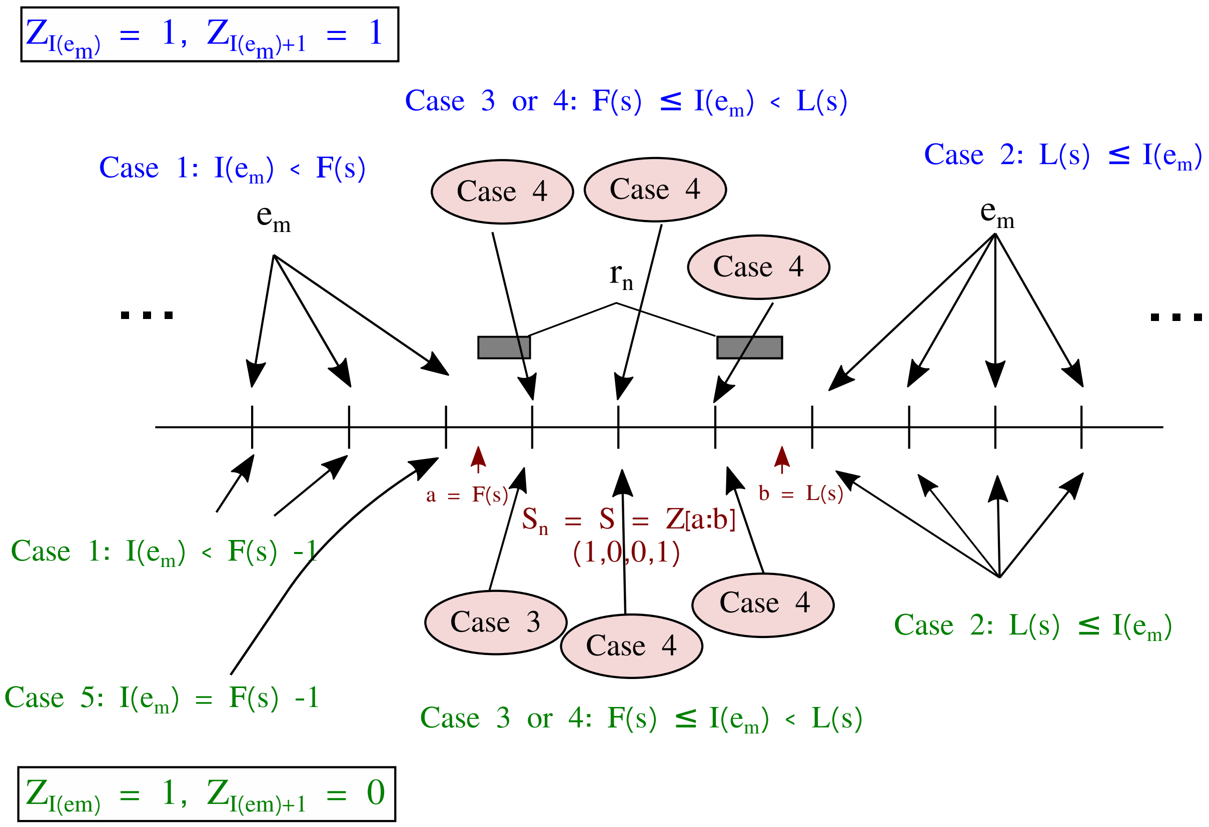

Figure S20: Visualization of the different cases considered for computing $\mathsf{P}(Z^n_{I(e_m)} = 1, Z^n_{I(e_m)+1} = 1, S_n = s)$ in the upper part, and $\mathsf{P}(Z^n_{I(e_m)} = 1, Z^n_{I(e_m)+1} = 0, S_n = s)$ in the lower part. Arrows from each case point to an exon end site or a set of exon end sites. a and b represent the indices of the first and last segments of the subpath $S_n = s$ from which read r_n is derived. In the upper part for $\mathsf{P}(Z^n_{I(e_m)} = 1, Z^n_{I(e_m)+1} = 1, S_n = s)$, an exon end site e_m appears to the left of subpath s (case 1), to the right of s (case 2), or within s (case 4). We do not allow for case 4 where $Z^n_{I(e_m)} = 1$, $Z^n_{I(e_m)+1} = 1$ are not compatible with subpath s. In the lower part for $\mathsf{P}(Z^n_{I(s_m)} = 1, Z^n_{I(s_m)+1} = 0, S_n = s)$, s_m appears to the left of s (cases 1 and 5), to the right of s (case 2), or within s (cases 3 and 4). We do not allow for cases 4 and 5 where $Z^n_{I(s_m)} = 1$ and $Z^n_{I(s_m)+1} = 0$ are not compatible with subpath s.

2.5.8.2 case 2: $L(s) \leq I(s_m)$

As shown in Figure S20, e_m appears right side of a subpath $s = z_{[a:b]}(o_a, \ldots, o_b)$.

$$
\begin{aligned}
&\mathsf{P}(S = s, Z_{I(e_m)} = 1, Z_{I(e_m)+1} = 1) \\
&= \mathsf{P}(Z_1 = 0, S = s, Z_{I(e_m)} = 1, Z_{I(e_m)+1} = 1) + \mathsf{P}(Z_1 = 1, S = s, Z_{I(e_m)} = 1, Z_{I(e_m)+1} = 1) \\
&= (1-\pi) f_{01}(1, F(s)) w(s) f_{11}(L(s), I(e_m))(1 - q_m) + \pi f_{11}(1, F(s)) w(s) f_{11}(L(s), I(e_m))(1 - q_m) \\
&= \left[(1-\pi) f_{01}(1, F(s)) + \pi f_{11}(1, F(s))\right] \times w(s) f_{11}(L(s), I(e_m))(1 - q_m),
\end{aligned} \tag{88}
$$

where $f_{..}(i, j)$ and $w(s)$ can be computed as described in Sections 2.5.4 and 2.5.5.

2.5.8.3 case 3: $F(s) \leq I(e_m) < L(s)$ and $(Z_{I(e_m)} = 1, Z_{I(e_m)+1} = 1)$ is a subset of s

As shown in Figure S20, e_m appears inside of a subpath $s = z_{[a:b]}(o_a, \ldots, o_b)$.

$$
\begin{aligned}
&\mathsf{P}(S = s, Z_{I(e_m)} = 1, Z_{I(e_m)+1} = 1) \\
&= \mathsf{P}(S = s) \\
&= \mathsf{P}(Z_1 = 0, S = s) + \mathsf{P}(Z_1 = 1, S = s) \\
&= (1-\pi) f_{01}(1, F(s)) w(s) + \pi f_{11}(1, F(s)) w(s) \\
&= \left[(1-\pi) f_{01}(1, F(s)) + \pi f_{11}(1, F(s))\right] \times w(s),
\end{aligned} \tag{89}
$$

where $f_{..}(i, j)$ and $w(s)$ can be computed as described in Sections 2.5.4 and 2.5.5.

2.5.8.4 case 4: $F(s) \leq I(e_m) < L(s)$ and $(Z_{I(e_m)} = 1, Z_{I(e_m)+1} = 1)$ is not a subset of s

In this case, $Z_{I(e_m)} = 1$ and $Z_{I(e_m)+1} = 1$ are not compatible with subpath s.

$$
\mathsf{P}(S = s, Z_{I(e_m)} = 1, Z_{I(s_m)+1} = 1) = 0 \tag{90}
$$

2.5.9 Computation of $\mathsf{P}(Z^n_{I(e_m)} = 1, Z^n_{I(e_m)+1} = 0, S_n = s)$

The E-step in Section 2.4.2 used $\mathsf{P}(Z^n_{I(e_m)} = 1, Z^n_{I(e_m)+1} = 0, S_n = s)$. Here, we describe how to compute these probabilities for the different cases when an exon end site e_m appears to the left, to the right, or within a subpath s. Figure S20 illustrates the different cases. As these quantities are identical for all reads r_n, we drop index n in this section for simplicity.

2.5.9.1 case 1: $I(e_m) < F(s) - 1$

As shown in Figure S20, an exon end site e_m appears left side of a subpath $s = z_{[a:b]}(o_a, \ldots, o_b)$.

$$
\begin{aligned}
&\mathsf{P}(Z_{I(e_m)} = 1, Z_{I(e_m)+1} = 0, S = s) \\
&= \mathsf{P}(Z_1 = 0, Z_{I(e_m)} = 1, Z_{I(e_m)+1} = 0, S = s) + \mathsf{P}(Z_1 = 1, Z_{I(e_m)} = 1, Z_{I(e_m)+1} = 0, S = s) \\
&= (1-\pi) f_{01}(1, I(e_m)) q_m f_{01}(I(s_m) + 1, F(s)) w(s) + \pi f_{11}(1, I(s_m)) q_m f_{01}(I(s_m) + 1, F(s)) w(s) \\
&= \left[(1-\pi) f_{01}(1, I(e_m)) + \pi f_{11}(1, I(s_m))\right] \times q_m f_{01}(I(s_m) + 1, F(s)) w(s),
\end{aligned} \tag{91}
$$

where $f_{..}(i, j)$ and $w(s)$ can be computed as described in Sections 2.5.4 and 2.5.5.

2.5.9.2 case 2: $L(s) \leq I(e_m)$

As shown in Figure S20, e_m appears right side of a subpath $s = z_{[a:b]}(o_a, \ldots, o_b)$.

$$\begin{aligned}
&\mathsf{P}(S = s, Z_{I(e_m)} = 1, Z_{I(e_m)+1} = 0) \\
&= \mathsf{P}(Z_1 = 0, S = s, Z_{I(e_m)} = 1, Z_{I(e_m)+1} = 0) + \mathsf{P}(Z_1 = 1, S = s, Z_{I(e_m)} = 1, Z_{I(e_m)+1} = 0) \\
&= (1-\pi) f_{01}(1, F(s)) w(s) f_{11}(L(s), I(s_m)) q_m + \pi f_{11}(1, F(s)) w(s) f_{11}(L(s), I(s_m)) q_m \\
&= \left[(1-\pi) f_{01}(1, F(s)) + \pi f_{11}(1, F(s))\right] \times w(s) f_{11}(L(s), I(s_m)) q_m,
\end{aligned} \tag{92}$$

where $f_{\cdot\cdot}(i, j)$ and $w(s)$ can be computed as described in Sections 2.5.4 and 2.5.5.

2.5.9.3 case 3: $F(s) \leq I(e_m) < L(s)$ and $(Z_{I(e_m)} = 1, Z_{I(e_m)+1} = 0)$ is a subset of s

As shown in Figure S20, e_m appears inside of a subpath $s = z_{[a:b]}(o_a, \ldots, o_b)$.

$$\begin{aligned}
&\mathsf{P}(S = s, Z_{I(e_m)} = 1, Z_{I(e_m)+0} = 1) \\
&= \mathsf{P}(S = s) \\
&= (1-\pi) f_{01}(1, F(s)) w(s) + \pi f_{11}(1, F(s)) w(s) \\
&= \left[(1-\pi) f_{01}(1, F(s)) + \pi f_{11}(1, F(s))\right] \times w(s),
\end{aligned} \tag{93}$$

where $f_{\cdot\cdot}(i, j)$ and $w(s)$ can be computed as described in Sections 2.5.4 and 2.5.5.

2.5.9.4 case 4: $F(s) \leq I(e_m) < L(s)$ and $(Z_{I(e_m)} = 1, Z_{I(e_m)+1} = 0)$ is not a subset of s

In this case, $Z_{I(e_m)} = 1$ and $Z_{I(e_m)+1} = 0$ are not compatible with subpath s.

$$\mathsf{P}(Z_{I(e_m)} = 1, Z_{I(e_m)+1} = 0, S = s) = 0 \tag{94}$$

2.5.9.5 case 5: $I(e_m) = F(s) - 1$

In this case, $Z_{I(e_m)} = 1$ and $Z_{I(e_m)+1} = 0$ are not compatible with subpath s.

$$\mathsf{P}(Z_{I(e_m)} = 1, Z_{I(e_m)+1} = 0, S = s) = 0. \tag{95}$$

2.5.10 Computation of $\mathsf{P}(Z_1^n = 1, S_n = s)$ and $\mathsf{P}(Z_1^n = 0, S_n = s)$

The E-step in Section 2.4.2 used $\mathsf{P}(Z_1^n = 1, S_n = s)$ and $\mathsf{P}(Z_1^n = 0, S_n = s)$. Here, we describe how to compute these probabilities. As these quantities are identical for all reads r_n, we drop index n in this section for simplicity.

$$\begin{aligned}
\mathsf{P}(Z_1 = 1, S = s) &= \mathsf{P}(Z_1 = 1)\mathsf{P}(Z_{F(s)} = 1 | Z_1 = 1)\mathsf{P}(S = s | Z_{F(s)} = 1) \\
&= \pi f_{11}(1, F(s)) w(s),
\end{aligned} \tag{96}$$

and

$$\begin{aligned}
\mathsf{P}(Z_1 = 0, S = s) &= \mathsf{P}(Z_1 = 0)\mathsf{P}(Z_{F(s)} = 1 | Z_1 = 0)\mathsf{P}(S = s | Z_{F(s)} = 1) \\
&= (1-\pi) f_{01}(1, F(s)) w(s),
\end{aligned} \tag{97}$$

where $f_{\cdot\cdot}(i, j)$ and $w(s)$ can be computed as described in Sections 2.5.4 and 2.5.5.

2.6 Benchmarks

2.6.1 Tools and parameters

2.6.1.1 Polyester simulator

We used simulated data to evaluate McSplicer accuracy. As mentioned in the main text, we used Polyester simulator (version 1.16.0) to simulate RNA-seq reads from human transcripts (Ensembl release 91). For the three different sequencing depths, we used the software with its default parameters, and we ran it under the following environment:

```
R version 3.5.2 (2018-12-20)
Platform: x86_64-redhat-linux-gnu (64-bit)
Running under: Scientific Linux 7.5 (Nitrogen)
```

As previously mentioned, we provided Polyester with ground truth abundances computed by running RSEM quantification tool [6] on RNA-seq data obtained from SRA data set SRR6987574 [2]. Then, we randomly selected a set of 1000 genes which have at least two expressed transcripts and have sufficiently high expression, i.e., gene-level read count per kilobase > 500.

2.6.1.2 STAR aligner

The simulated reads were mapped to the human reference genome (GRCh38.91) by running STAR (version 2.5.4b) [1] with the following parameters:

```
--runMode alignReads
--outSAMtype BAM SortedByCoordinate
--sjdbGTFfile Homo_sapiens.GRCh38.91.gtf
--runThreadN 16
--readFilesIn {ployester_output.fasta}
--outFileNamePrefix {output_prefix}
--genomeDir {genome_directory}
--outSAMstrandField intronMotif
--sjdbGTFfile
```

The remaining set of parameters were left to the default values.

For indexing the resulting BAM files we used Samtools (version 0.1.8) [7].

2.6.1.3 StringTie

We ran StringTie [8] (version 1.3.4d) with genome-guided mode enabled (-G option) and provided Ensembl annotation release 91. The remaining parameters of StringTie were left to the default values.

2.6.1.4 SplAdder

We ran SplAdder (version 1.2.0) with the following set of parameters for benchmarking on simulated data:

[2] http://www.ncbi.nlm.nih.gov/sra

```
--bams {bam_files}
--annotation {annotation_gtf}
--merge_strat merge_graphs
--event_types exon_skip,intron_retention,alt_3prime,
alt_5prime,mult_exon_skip
--confidence 2
--pyproc n
--compress_text n
--ignore_mismatches y
--outdir {output_directory}
```

We set the *confidence* parameter to 1 when running SplAdder on the SIRV dataset in order to detect novel events.

2.6.1.5 MAJIQ

We ran MAJIQ (version 2.0) with default parameters but with de novo option disabled, i.e., $disable-denovo$ for all benchmarks on simulated data. We noticed many false positive events when running MAJIQ without the $disable-denovo$ argument (i.e., enabling de novo mode). We enable de novo mode again when evaluating MAJIQ on SIRV data sets to detect as many novel events as possible.

2.6.1.6 PSGInfer

To compute edge weight estimates using PSGInfer, we followed two steps. First, we executed the command psg_prepare_reference to generate a reference splice graph from annotated transcripts (Ensembl annotation release 91), and we configured it to generate a line graph since it is computationally more efficient than other types of graphs yet provides accurate estimates of edge weights [5]. Second, we ran psg_infer_frequencies to map RNA-seq reads to the splice graphs generated in the first step and to estimate the weights of graph edges. PSGInfer uses Bowtie [3] internally for RNA-seq read mapping. We ran the latest version of PSGInfer 1.2.1 and a compatible version of Bowtie 1.3.0.

```
--annotations {annotation_gtf}
--genome-dir {chromosome_FASTA_files_dir}
-l 100 {max_read_length}
-k 0 {order_of_PSG}
--num-threads 72
```

2.6.2 Comparable splice sites

Let $s_1, s_2, \ldots, s_{M_G}$ denote the splice sites and transcription start and end sites of a gene G, ordered by their genomic coordinates. Consistent with [2], we define *alternative splicing events* for pairs of expressed transcripts t_1, t_2 as maximal sequences $s_i, \ldots, s_j$ of alternative splice sites, i.e. splice sites that are used by t_1 or by t_2, but not by both. To distinguish the outcome of alternative splicing from the outcome of alternative transcription initiation or termination, we additionally require that s_{i-1} and s_{j+1} denote common donor and acceptor sites, respectively. If every transcript expressed by G is consistent with t_1 or t_2 in its use of $s_{i-1}, s_i, \ldots, s_{j+1}$, we call the alternative splice sites $s_i, \ldots, s_j$ *comparable*. Note that the

definition of comparable splice sites is invariant with respect to the choice of t_1 and t_2 among expressed transcripts of G.

2.6.3 True splice site usage

Let $A(s)$ and $B(s)$ denote subsets of transcripts in a gene G that either use or do not use a particular splice site s, respectively. Then the true usage of splice site s is computed by

$$u_s = \frac{\sum_{t\in A(s)} \theta_t}{\sum_{t\in A(s)\cup B(s)} \theta_t} = \frac{\sum_{t\in A(s)} \theta_t}{\sum_{t\in G} \theta_t}, \tag{98}$$

where θ_t represents the true abundance of transcript t.

2.6.4 Kullback-Leibler (KL) divergence

For a given splice site s, the two possible outcomes, whether or not a transcript uses the splice site can be modelled by a Bernoulli distribution with the splice site usage u_s, denoted by Bernoulli(u_s). Let $\hat{u}_s$ represent the estimated splice usage. Then, we measure the accuracy of $\hat{u}_s$ using the KL divergence of Bernoulli$(\hat{u}_s)$ from Bernoulli(u_s):

$$D_{KL}(\text{Bernoulli}(u_s)||\text{Bernoulli}(\hat{u}_s)) = u_s \log\frac{u_s}{\hat{u}_s} + (1-u_s)\log\frac{1-u_s}{1-\hat{u}_s}. \tag{99}$$

References

[1] Alexander Dobin, Carrie A Davis, Felix Schlesinger, Jorg Drenkow, Chris Zaleski, Sonali Jha, Philippe Batut, Mark Chaisson, and Thomas R Gingeras. Star: ultrafast universal rna-seq aligner. *Bioinformatics*, 29(1):15–21, 2013.

[2] Sylvain Foissac and Michael Sammeth. Astalavista: dynamic and flexible analysis of alternative splicing events in custom gene datasets. *Nucleic acids research*, 35(suppl_2):W297–W299, 2007.

[3] Ben Langmead, Cole Trapnell, Mihai Pop, and Steven L Salzberg. Ultrafast and memory-efficient alignment of short dna sequences to the human genome. *Genome biology*, 10(3):R25, 2009.

[4] Laura H LeGault and Colin N Dewey. Inference of alternative splicing from rna-seq data with probabilistic splice graphs. *Bioinformatics*, 29(18):2300–2310, 2013.

[5] Laura H LeGault and Colin N Dewey. Inference of alternative splicing from rna-seq data with probabilistic splice graphs. *Bioinformatics*, 29(18):2300–2310, 2013.

[6] Bo Li and Colin N Dewey. Rsem: accurate transcript quantification from rna-seq data with or without a reference genome. *BMC bioinformatics*, 12(1):323, 2011.

[7] Heng Li, Bob Handsaker, Alec Wysoker, Tim Fennell, Jue Ruan, Nils Homer, Gabor Marth, Goncalo Abecasis, and Richard Durbin. The sequence alignment/map format and samtools. *Bioinformatics*, 25(16):2078–2079, 2009.

[8] Mihaela Pertea, Geo M Pertea, Corina M Antonescu, Tsung-Cheng Chang, Joshua T Mendell, and Steven L Salzberg. Stringtie enables improved reconstruction of a transcriptome from rna-seq reads. *Nature biotechnology*, 33(3):290, 2015.

[9] Cole Trapnell, Brian A. Williams, Geo Pertea, Ali Mortazavi, Gordon Kwan, Marijke J. van Baren, Steven L. Salzberg, Barbara J. Wold, and Lior Pachter. Transcript assembly and quantification by rna-seq reveals unannotated transcripts and isoform switching during cell differentiation. *Nature biotechnology*, 28(5):511–515, May 2010. 20436464[pmid].
